# In Situ Assembly of Fluorogenic RNA for Screening Natural Anti‐Liver Fibrosis Products via Dynamic Visualization of *COL1A1* mRNA

**DOI:** 10.1002/advs.202502850

**Published:** 2025-05-23

**Authors:** Rui Bai, Li‐Zeng Zhu, Changfa Shao, Zheng Yin, Qun Liu, Yu Gu, Bin Liu

**Affiliations:** ^1^ School of Materials Science and Engineering Suzhou University of Science and Technology Kerui Road Suzhou 215009 P. R. China; ^2^ State Key Laboratory of Natural Medicines School of Traditional Chinese Pharmacy China Pharmaceutical University Nanjing 210009 P. R. China; ^3^ Department of Chemical and Biomolecular Engineering National University of Singapore Singapore 117585 Singapore

**Keywords:** anti‐liver fibrosis, aptamers, dynamic visualization, imaging, mRNA

## Abstract

Liver fibrosis, a critical precursor to cirrhosis and a leading cause of mortality, highlights the urgent need for the identification of effective therapeutics. Activation of hepatic stellate cells (HSCs) is a key process in liver fibrosis. This study presents a live‐cell drug‐screening approach that specifically targets fibrosis‐associated collagen type I alpha 1 (*COL1A1*) mRNA in activated HSCs through the use of fluorogenic RNA self‐assembly. It employs a dual‐probe system to construct an RNA Mango II structure, which upon binding with *COL1A1* mRNA, facilitates activation of the TO1‐Biotin fluorophore, thereby enabling the visualization of mRNA to indicate HSC activation levels. Through the application of a high throughput live‐cell screening system, dihydrotanshinone I (DHT) is identified as potent leading antifibrotic compound, evidenced by its inhibitory effects on *COL1A1* mRNA expression. The therapeutic efficacy of DHT is further substantiated by monitoring *COL1A1* mRNA dynamics following treatment. In *vivo* studies demonstrates the sustained administration of DHT significantly ameliorated liver fibrosis in mice models. This method offers a simple, cost‐effective approach of visualizing RNA dynamics and conducting drug screening in live cells, presenting a significant potential for the development of hepatic fibrosis therapies.

## Graphical abstract

This study introduces an in situ assembly of fluorogenic RNA approach for screening natural anti‐liver fibrosis compounds and dynamic visualization of endogenous *COL1A1* mRNA in live cells. It offers a simple, cost‐effective approach of visualizing RNA dynamics and conducting drug screening in live cells, presenting a significant potential for the development of hepatic fibrosis therapies.

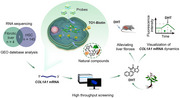

## 1 Introduction

Liver fibrosis is a pathological condition characterized by the excessive accumulation of extracellular matrix (ECM) proteins, primarily collagen, within the liver.^[^ ^1^ ^]^ This fibrous tissue buildup is a response to chronic liver injury from various causes, such as viral infections, alcohol abuse, autoimmune diseases, or metabolic dysfunction‐associated steatohepatitis (MASH). If left untreated, persistent liver fibrosis can progress to severe liver cirrhosis, hepatocellular carcinoma, or even death. The incidence of advanced liver fibrosis and its complications is nearly 5 per 100 person‐years, posing a significant threat to human health.^[^ ^2^ ^]^ Currently, FDA has approved only one drug for liver fibrosis, Rezdiffra, a thyroid hormone receptor‐beta agonist, which targets MASH‐related fibrosis.^[^ ^3^ ^]^ The limited number of drug options and the narrow applicability of the available therapy indicate the clear shortage of anti‐liver fibrosis medications. In addition, the fibrous deposition in hepatic fibrosis is reversible in its initial stage, making it crucial for preventing liver cirrhosis.^[^ ^4^ ^]^ This highlights the substantial unmet need for a deeper understanding of the fibrogenesis process and the development of effective therapeutic strategies to prevent liver failure.

The activation and proliferation of HSCs are critical events in liver fibrosis.^[^ ^5^ ^]^ When HSCs are exposed to injurious stimuli, they undergo a phenotypic change to an activated state, leading to excessive ECM deposition and liver function impairment.^[^ ^6^ ^]^ Monitoring key molecular targets associated with HSC activation and proliferation is essential for assessing therapeutic interventions, with most anti‐fibrotic strategies, including immunofluorescence^[^ ^7^ ^]^ and luciferase assays in combination with quantitative real‐time polymerase chain reaction (qRT‐PCR) analysis^[^ ^8^ ^]^ fundamentally relying on these principles. The immunofluorescence‐based screening method involves the visualization of fibrocyte activation proteins such as fibronectin (FN)^[^ ^7^ ^]^ and alpha smooth muscle actin (alpha‐SMA)^[^ ^9^, ^10^ ^]^ through their high‐specificity binding with fluorescently labeled antibodies, allowing for the assessment of activation level of HSCs only in fixed cells. In addition, the high cost of antibody procurement rules out its use in high throughput drug screening initiatives. Luciferase reporter assays^[^ ^11^ ^]^ are valuable for studying gene regulation and screening compounds that modulate gene expression.^[^ ^8^ ^]^ However, they are limited by the need for cell lysis to measure luciferase activity, which prevents real‐time analysis of live‐cell dynamics. Direct drug screening in live cells provides a more precise insight of their behavior within natural cellular environment and mitigates the negative impacts of cell lysis and morphological alterations.^[^ ^12^ ^]^ Therefore, it is crucial to develop an affordable, user‐friendly drug‐screening method for live cells. Fortunately, during HSC activation, specific mRNA expressions^[^ ^13^, ^14^ ^]^ are upregulated, and precise targeting and downregulating these mRNAs could potentially prevent or reverse the fibrotic process. This implies that establishing a general in situ mRNA imaging method in fibroblasts to visualize the degree of HSC activation is highly beneficial in the search for effective antifibrotic therapies.

CRISPR (Clustered Regularly Interspaced Short Palindromic Repeats)‐Cas13 system^[^ ^15^ ^]^ and MS2 analogous systems, like MS2: MCP (MS2 coat protein),^[^ ^16^ ^]^ PP7: PCP (PP7 coat protein),^[^ ^17^ ^]^ boxB: λN_22_,^[^ ^18^ ^]^ are current widely used methods to investigate intracellular mRNA dynamic in live cells. Nevertheless, MS2‐like systems suffer primarily from the need to artificially modify the target RNA, thereby unsuitable for endogenous RNA imaging. CRISPR‐Cas13 avoids modifying the target RNA but faces challenges with potential perturbation by the Cas13 protein itself, which could interfere with native RNA‐protein interactions, RNA translation or degradation. Despite compact structure, ease of synthesis, and enhanced sensitivity, molecular beacon (MB) probes^[^ ^19^ ^]^ require precise dual‐labeling (fluorophore‐quencher conjugation) on oligonucleotides, a technically demanding process that increase costs and limits their use in high‐throughput sample analyses. Fluorogenic RNA aptamers, which selectively bind to and activate the fluorescence of nonfluorescent fluorophores, have garnered global interest due to their compact sequences, facile programming, and minimal cellular background. Over the past decade, various RNA aptamers, including the Spinach family,^[^ ^20^ ^]^ Broccoli,^[^ ^21^ ^]^ Corn,^[^ ^22^, ^23^ ^]^ Mango,^[^ ^24^ ^]^ Pepper,^[^ ^25^ ^]^ Okra,^[^ ^26^ ^]^ and Squash,^[^ ^27^ ^]^ have been developed to image cellular RNA species with a low background signal. In these systems, RNA aptamers are usually conjugated to the ends of RNA through genetic encoding, such as inserting RNA aptamer sequences into the 3'UTRs of target mRNA for cellular expression. Although this design offers high specificity and low background, it requires the modification of RNA transcripts, thus only allowing the visualization of engineered, exogenous mRNAs in live cells, but not endogenous mRNAs.^[^ ^28^ ^]^ Direct imaging of non‐engineered, endogenous mRNAs with RNA aptamers remains challenging. Fan et al. provided a powerful approach named RNA aptamer‐triggered fluorescence complementation for in situ labeling of β‐actin mRNA in HeLa cells with split Broccoli.^[^ ^29^ ^]^ To address the low sensitivity issue, Liu's group developed nanozippers that assemble into a triad of Broccoli upon target recognition, facilitating real‐time imaging of the dynamic translation of individual viral mRNAs.^[^ ^30^ ^]^ However, the Spinach family or Broccoli‐bound DFHBI experiences a rapid decrease in fluorescence due to light‐induced isomerization of DFHBI,^[^ ^31^, ^32^ ^]^ hindering long‐term measurements. The well‐known Mango II aptamer, which binds with high affinity to thiazole orange Biotin conjugate (TO1‐Biotin), has enabled single‐molecule exogenous mRNA tracking in live cells with better photostability than DFHBI‐binding aptamers.^[^ ^24^ ^]^ To effectively observe the dynamic biological activities involving mRNA within live cells and thoroughly investigate the changes in mRNA and their underlying mechanisms following drug stimulation, it is crucial to accurately locate and track endogenous mRNA within the cellular environment.

**Scheme 1:**
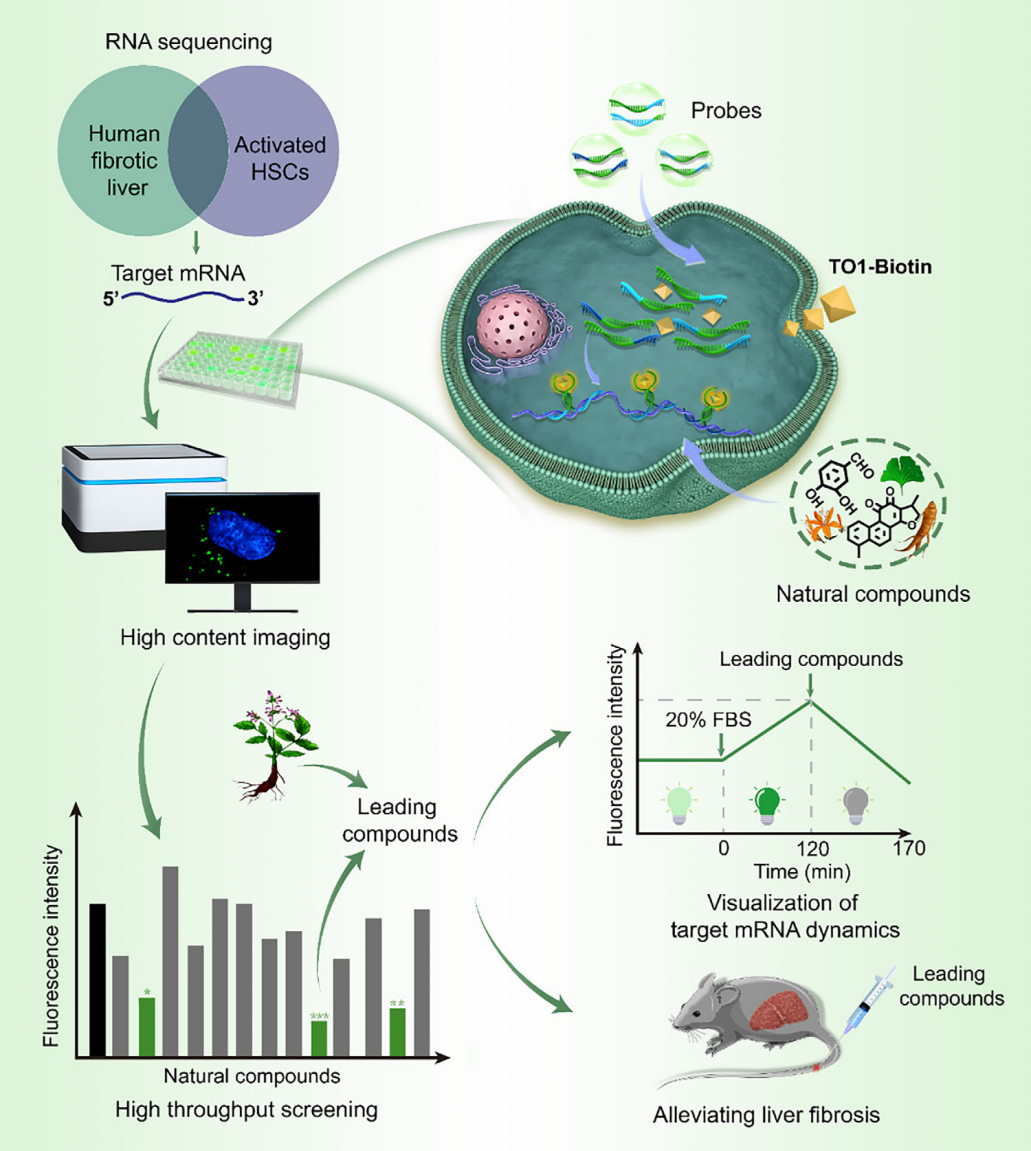
Schematic illustration of target‐induced fluorogenic RNA self‐assembly for high throughput screening of anti‐liver fibrosis natural products in live cells.

Herein, we designed RNA recognition probes based on the fluorogenic RNA self‐assembly and successfully applied these probes in measuring endogenous mRNA dynamics and drug screening (**Scheme** **1**). Target mRNA, reflecting the activation levels of HSCs, was selected through an integrative analysis of both clinical and non‐clinical data on Gene Expression Omnibus (GEO) obtained from the National Center for Biotechnology Information (NCBI) database. In the presence of target mRNA, probes self‐assemble into a Mango II structure that specifically binds to TO1‐Biotin, triggering fluorescence and lighting‐up RNA target. Employing these probes, we developed live‐cell imaging system for the high throughput assessment of natural products' effects on target mRNA levels to alleviate liver fibrosis. The probes also facilitated confirmation of screening results through a 120 min time‐resolved mRNA kinetics analysis in HSCs post‐treatment with the leading compounds. Ultimately, the therapeutic potential of the screened compounds was validated in *vivo*, demonstrating their ability to reduce fibrosis in a CCl_4_‐induced mouse liver fibrosis model. This technique enables drug screening within live cells and the dynamic visualization of RNA, reducing the costs associated with drug screening and propelling the use of RNA aptamers in cellular imaging and drug discovery.

## 2 Results and Discussion

### 2.1 Design of Conformation‐Induced Fluorogenic RNA Self‐Assembly Probes

**Figure 1:**
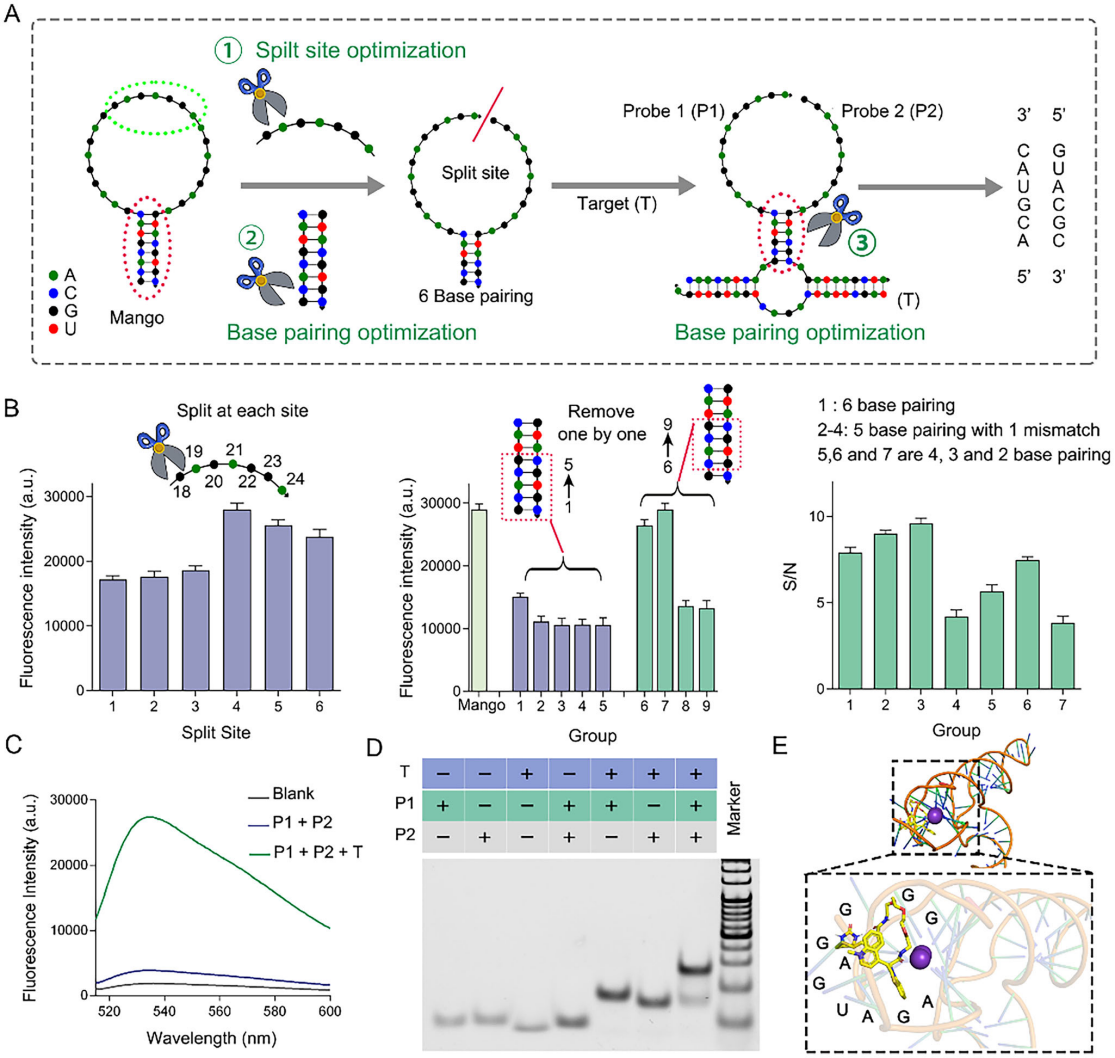
Probe optimization and validation. A) Schematic illustration of probe optimization process. B) Fluorescence intensity at 535 nm of probes incubated 2 h in HEPES buffer (40 mM, pH 7.4) containing 10 µM TO1‐Biotin, 100 mM KCl, and 10 mM MgCl_2_. C) Fluorescence emission spectrum of probes excited at 505 nm after incubating with or without T in HEPES buffer (40 mM, pH 7.4) containing 10 µM TO1‐Biotin, 100 mM KCl, and 10 mM MgCl_2_. D) Native‐PAGE results for the reaction of probes 1 and 2 either present or absent of T; the “+” denotes the addition of these strands. E) MD simulations of the binding sites of two probes after target recognition and TO1‐Biotin. The labeled bases are interacting with TO1‐Biotin molecules.

RNA Mango II is remarkable due to its resistance to formaldehyde fixation, enhanced thermal stability and strong binding capacity to TO1‐Biotin, with approximately 1 nM affinity and over 1500‐fold enhanced fluorescence. According to literature, the co‐crystal structure of RNA Mango II disclosed with a three‐tiered G‐quadruplex (T1, T2, and T3), and the binding site of TO1‐Biotin molecules attaches to the adenines in the four propeller loops and the planar T3 quartet of Mango II (Figure S1, Supporting Information).^[^ ^33^ ^]^ Based on this, we first split Mango II into two sequences from its adenines and T3 quartet (5’‐GAGAGGA‐3’), which is the key site for TO1‐Biotin binding (**Figure** **1**A①, Table S1, Supporting Information), and then determine its fluorescence intensity at 535 nm (excited at 505 nm, Figure 1b) after adding TO1‐Biotin. The two sequences can still hybridize without further modification as there are eight base complements (Delta G value: −13.69 kcal mol^−1^, *T* = 37 °C) by analyzing with Oligo Analyzer 3.1 software from Integrated DNA Technologies. It appears that the splitting between probes 1 (P1) and probe 2 (P2) at the 21^st^ of adenine and 22^nd^ of cytosine results in the highest fluorescence increase after being incubated with TO1‐Biotin. To reduce the binding strength between the two RNA sequences, we modified the number of complementary bases in the stem region accordingly (Figure 1A②). The results are shown in the middle of Figure 1B. When the bottom base pair domain of G‐C was removed, the fluorescence intensity decreased obviously, suggesting its importance in forming RNA Mango II structure for TO1‐Biotin binding. We then kept the bottom G‐C and removed base pairs one by one from the secondary C‐G, and the fluorescence after their binding with TO1‐Biotin decreased almost half when its stem length was less than 6 bp. In other words, the 6 base‐pairs stem was regarded as the equilibrium point for the two probes to achieve a functional structure. Based on the above results, we further optimized the neck unit by extending the two split probes with an 8 or 9‐base RNA arm that was complementary to an RNA target (T), separately. As shown in Figure 1B (right), the signal to noise ratio (S/N) keeps increasing until the base pairing of P1 and P2 is less than 5. The final length of stems after optimization is five‐base pairing as described in Figure 1A③.

To ensure that the two probes could accurately assemble into the structure of RNA Mango II in the presence of T and TO1‐Biotin, we measured the fluorescence spectra of these probes upon binding to TO1‐Biotin in the presence or absence of T. As shown in Figure 1C, TO1‐Biotin, as well as P1 incubated with P2, exhibit a low yet detectable background fluorescence, which is effective in preventing nonspecific signal leakage. Upon incubating P1 and P2 with T along with TO1‐Biotin, a significant increase in fluorescence intensity is observed with a peak emission wavelength of 535 nm under excitation at 505 nm. We further compared the original Mango II to the self‐assembly of P1 and P2 after incubation with the target, and the results are shown in Figure S2 (Supporting Information). Under identical conditions with a 1 µM concentration of P1 and P2, incubation with T and TO1‐Biotin, did not result in complete self‐assembly into 1 µM Mango II. The assembly efficiency was approximately 88.6%. It is noteworthy that 1 µM target concentration is significantly higher than typical intracellular RNA levels, therefore, P1 and P2 would predominantly remain in excess for intracellular RNA imaging. Notably, an RNA sequence with only a 1 nt mismatch to the target resulted in a 70% reduction in fluorescence, while a 2 nt mismatch led to an 82.1% decrease in fluorescence. This highlights the high selectivity and specificity of these probes (Figure S3 and Table S2, Supporting Information). The fluorescence emission spectra and fluorescence intensity at 535 nm of TO1‐Biotin, P1, and P2 (1 µM) in the presence of different target concentrations were monitored, and the results are presented in Figure S4 (Supporting Information). A gradual increase in fluorescence was observed as the concentration of T was increased. A linear correlation was found between fluorescence intensity and T concentration within the range of 0 to 1 µM, with a calculated detection limit of 1.23 nM for T.

The feasibility of the assemble process was also evaluated by performing polyacrylamide gel electrophoresis (PAGE). As depicted in Figure 1D, the band from a mixture of P1 and P2 fragments without T was comparable to that of the corresponding single probe. It is worth noticing that due to the very close molecular weights of P1 (28 nt) and P2 (27 nt), the band positions of the mixture of P1 and P2 fragments merged with a significant increase in brightness. However, when incubating T with P1 and P2, a new band with delayed mobility appeared, indicating hybridization between target RNA and the two probes. As P1 and P2 have complementary bases with T, hybridization also occurred when the target was incubated with either P1 or P2. However, this did not affect the selectivity of our system because T incubated with either P1 or P2 cannot offer TO1‐Biotin binding sites to activate its fluorescence enhancement (Figure 1C). Subsequently, Molecular Dynamics (MD) simulations were conducted to investigate the binding free energy and identify the binding sites of TO1‐Biotin molecules to the two probes following T recognition. As illustrated in Figure 1E, the structure of the two probes after T recognition has a preorganized binding pocket. The long axes of the benzothiazole and methylquinoline heterocycles within TO1‐Biotin are oriented perpendicular to those of the guanines stacked in the RNA complex. Circular dichroism spectra of RNA Mango II alone and our probes after incubating with target RNA were added to give evidence on the formation of Mango II structures after target recognition, and there are characteristic peaks of parallel G‐quadruplexes^[^ ^34^ ^]^ in both Mango‐ II and our probes after incubating with target RNA. The peaks’ wavelengths of our probes, following target recognition, exhibit a slight red shift compared to those of the Mango‐II aptamers, likely due to changes in their surrounding environment after RNA interactions (Figure S5, Supporting Information). Additionally, the adenines within the four propeller loops and the planar G‐quadruplex collectively offer the binding site of the fluorophore with a binding energy of ‐5.9 kcal mol^−1^. The results described above offer abundant information on the Mango II structural formed by our probes upon T identification, which hold potential for detecting endogenous mRNA in living cells.

### 2.2 Discovery of *COL1A1* mRNA as Specific Target of Liver Fibrosis by Gene Expression Omnibus (GEO) Database Analysis

**Figure 2:**
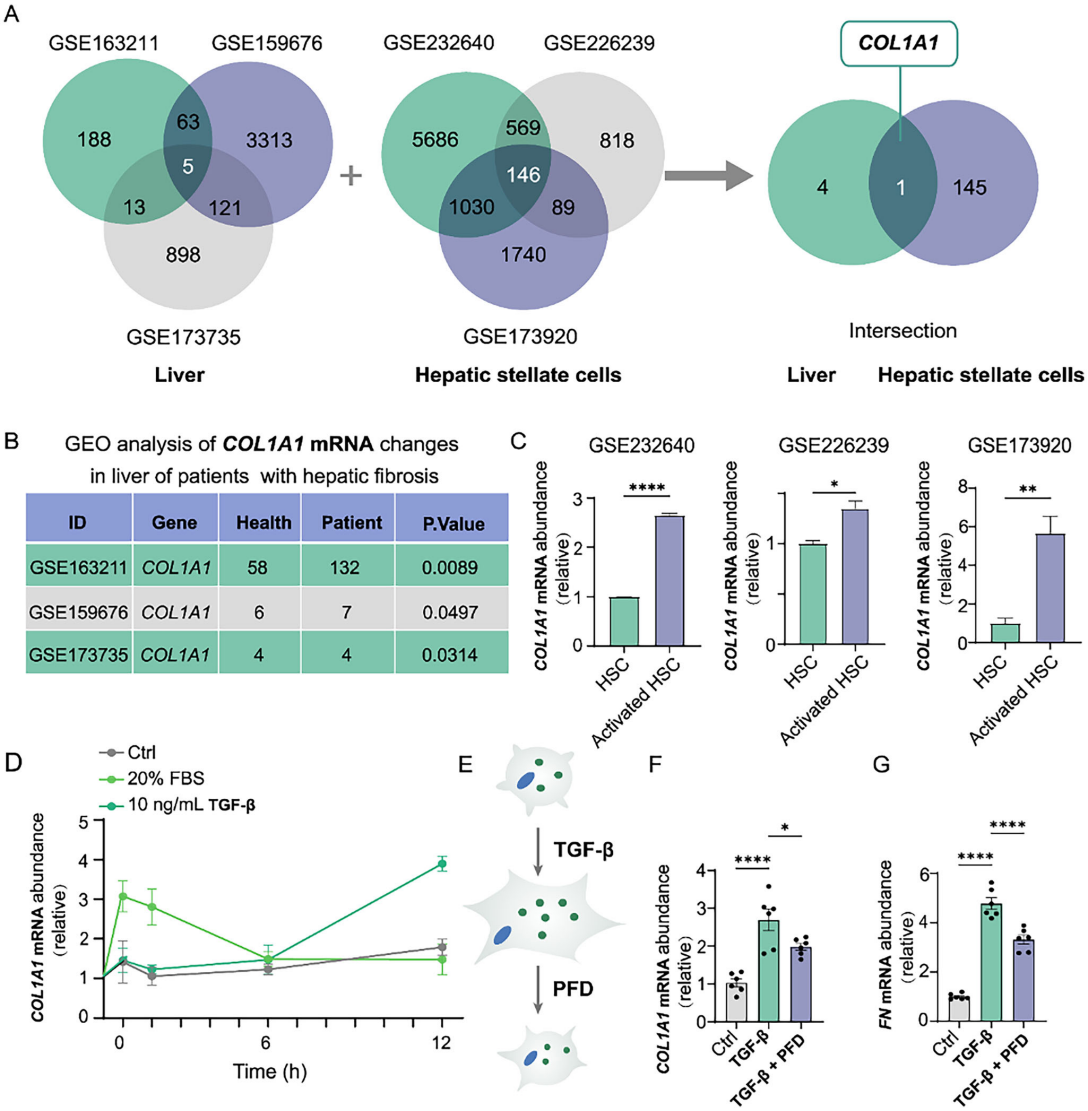
*COL1A1* mRNA emerges as a potential specific biomarker of liver fibrosis. A) Analysis of liver of hepatic fibrosis patients and activated HSCs from the GEO database. B) *COL1A1* mRNA changes in the liver of patients with hepatic fibrosis in the GSE163211, GSE159676, and GSE173735 datasets. C) *COL1A1* mRNA expression in the HSCs database of GSE232640 (n = 3), GSE226239 (n = 3), and GSE173920 (n = 3). D) Expression of *COL1A1* mRNA in LX‐2 cells was stimulated by treatment with 10 ng mL^−1^ of TGF‐β or 20% FBS (n = 6). E–G) mRNAs expression of *COL1A1* (F) and *FN* (G) in LX‐2 cells treated with TGF‐β (10 ng mL^−1^, 24 h) with or without 1 mM PFD (a TGF‐β inhibitor) (n = 6).

Recognizing that liver fibrosis is a widespread clinical disease with a multitude of unfavorable consequences, the mRNA genes that are liver fibrosis specific were then explored. Integrative analysis of clinical and non‐clinical data on GEO obtained from the NCBI database were used to identify the mRNAs most closely associated with the occurrence of liver fibrosis (**Figure** **2**A). The expression levels of mRNA in three liver fibrosis related GEO datasets, covering fibrotic liver tissue of 143 patients and 68 healthy individuals were first integrated, and then screened according to their expression correlation (*p* < 0.05).^[^ ^35^, ^36^ ^]^ The results showed that the expression of 5 mRNAs was significantly upregulated in non‐alcoholic steatohepatitis (GSE163211), non‐alcoholic fatty liver disease (GSE159676), non‐alcoholic steatohepatitis (GSE173735) liver tissues (Figure 2B). As activation of HSCs is a fundamental pathogenic event in liver fibrosis, the same method was used to screen out 146 kinds of significantly abnormally expressed mRNA in the activated HSCs (Figure 2C). Subsequently, *COL1A1* mRNA (Table S3, Supporting Information) was chosen as the primary target mRNA for further investigation due to its upregulated expression being closely associated with liver tissue from patients with liver fibrosis and activated HSCs.

The expression of *COL1A1* mRNA was also validated through qRT‐PCR analysis in human HSCs (LX‐2 cell line) with different treatment. To simulate the process of liver fibrosis, LX‐2 cells were activated by stimulation both of transforming growth factor‐β (TGF‐β)^[^ ^37^ ^]^ or 20% of serum,^[^ ^38^ ^]^ and the expression of *COL1A1* mRNA within 12 h was recorded through RT‐qPCR analysis. As illustrated in Figure 2D, the expression of *COL1A1* mRNA in HSCs was significantly upregulated in response to TGF‐β stimulation. Additionally, *COL1A1* mRNA level in LX‐2 cells after treated with TGF‐β reach a peak at 12h, followed by a plateau phase that was maintained through subsequent time points (18–24 hours, Figure S6, Supporting Information). Notably, the serum stimulation at a concentration of 20% exerted a markedly more potent effect during the first 2 h compared to TGF‐β stimulation in the activation of LX‐2 cells.

Pirfenidone (PFD), an anti‐fibrotic drug,^[^ ^39^ ^]^ inhibits the synthesis and activation of TGF‐β.^[^ ^13^ ^]^ Our study explored the correlation between *COL1A1* mRNA expression and HSC activation using TGF‐β for induction and PFD for inhibition (Figure 2E). *COL1A1* mRNA levels increased with TGF‐β treatment and decreased significantly with PFD treatment (Figure 2F). The activation of LX‐2 cells was also assessed by monitoring *FN* mRNA expression, a gene synthesized by activated HSCs early in TGF‐β‐induced fibrotic responses.^[^ ^7^ ^]^ The results in Figure 2G are consistent with *COL1A1* mRNA expression, indicating a positive correlation between *COL1A1* expression and HSC activation in LX‐2 cells. Notably, while *FN* mRNA upregulation was more pronounced than *COL1A1* mRNA upon TGF‐β stimulation of HSCs, *FN* was not considered for further investigation as it did not rank among the top 5 mRNAs with significant expression differences in human fibrotic liver. Meanwhile, we summarized the relevant studies regarding the use of *COL1A1* mRNA as a biomarker for liver fibrosis,^[^ ^40^, ^41^ ^]^ and *COL1A1* mRNA expression increased with disease progression of liver fibrosis (p < 0.001). Elevated expression levels of *COL1A1* mRNA were also observed in the livers of mice with hepatic fibrosis induced by carbon tetrachloride (CCl_4_), bile duct ligation (BDL), and thioacetamide (TAA), respectively. Thus, *COL1A1* mRNA emerges as a potential biomarker in the liver fibrosis process, which is also consistent with the literature.

### 2.3 RNA Probes for Labeling *COL1A1* mRNA in Cells

**Figure 3:**
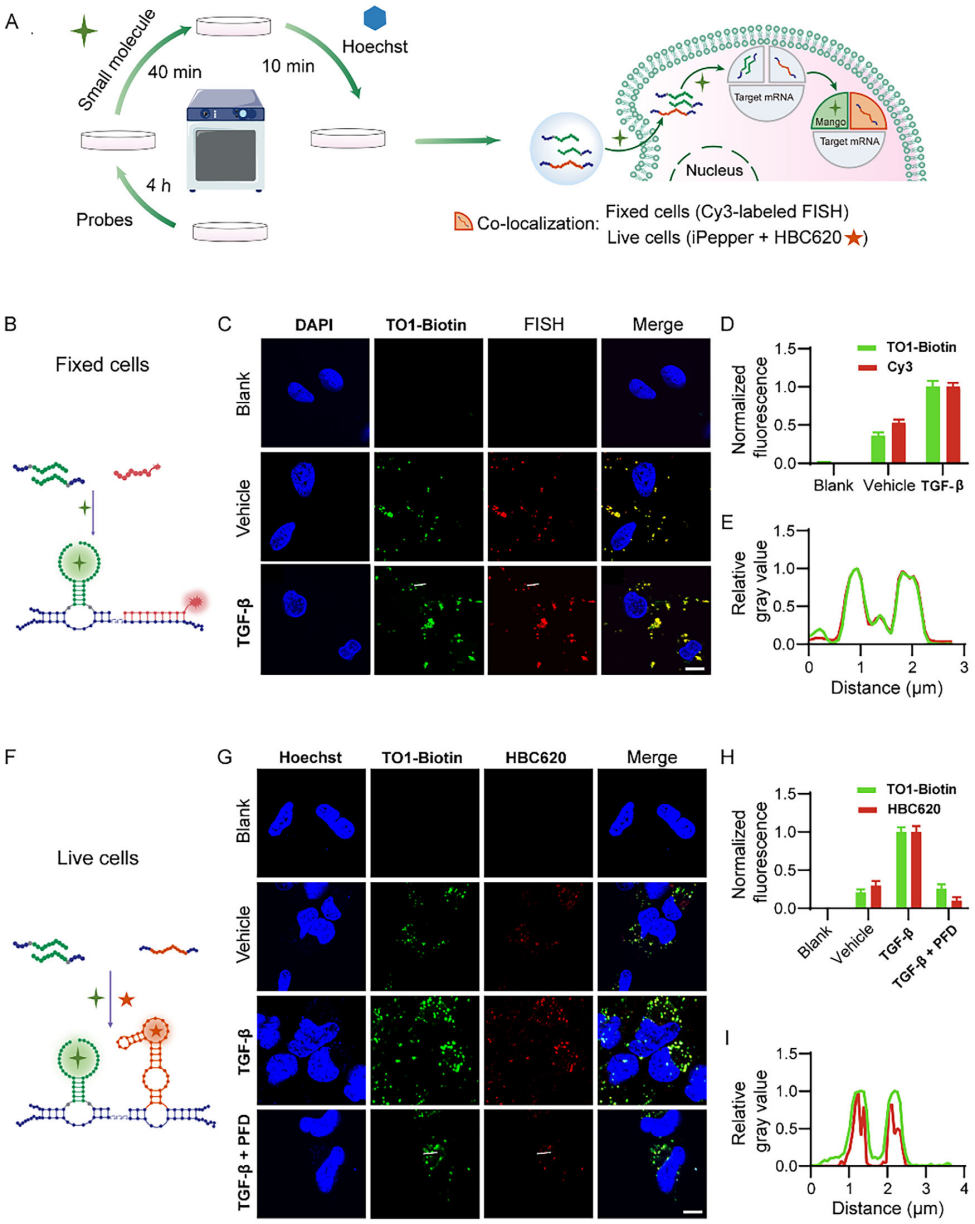
RNA probes for visualization of *COL1A1* mRNA in LX‐2 cells. A) Schematic representation of the co‐localization experiment using small molecules and probes. Fixed cells were imaged using Cy3‐labeled FISH and live cells were imaged using the iPepper probes and HBC620. B) Schematic illustration of probes binding with target mRNA in the fixed LX‐2 cells. C) Fixed‐cell co‐localization confocal images of transcribed probes with TO1‐Biotin (green) and Cy3‐labeled FISH (red) targeted *COL1A1* mRNA in LX‐2 cells. Scale bar = 5 µm. D) Fluorescence statistical histogram analysis of these cells calculated from confocal images in (C). E) The changes in relative fluorescence intensity along the straight line marked in the partial image in (C). F) Schematic of the probes binding with *COL1A1* mRNA in live LX‐2 cells. G) Live cells co‐localization confocal images of transcribed probes with TO1‐Biotin (green) and ipepper‐HBC620 (red) targeted *COL1A1* mRNA in LX‐2 cells. Scale bar = 5 µm. H) Fluorescence statistical histogram analysis of these cells calculated from confocal images in (G). I) The change in relative fluorescence intensity along the straight line marked in the partial image in (G).

As the variation of *COL1A1* mRNA level was selected to characterize the activation of HSCs, we proceeded to develop probes for visualizing *COL1A1* mRNAs (Table S3, Supporting Information) in human HSCs (**Figure** **3**A). The recognition unit was redesigned into the two fragmented parts derived from a specific 25 nt sequence (T1, Table S4, Supporting Information) of the entire *COL1A1* mRNA, with each fragment being extended by probes containing optimized split probes for TO1‐Biotin binding and activation. Equal amounts of these two probes (P1‐1 and P2‐1) and a co‐localization probe (Table S5, Supporting Information) were co‐transfected into LX‐2 cells. Following a 4 h transfection period, the cells were cultured with TO1‐Biotin for an additional 40 min before subsequent utilization (Figure 3A). The fluorescence intensity demonstrated a strong specific response for *COL1A1* mRNA compared to the other variants such as other collagen isoforms, including *COL1A2* and *COL1A3*, as well as fibrosis‐related transcripts like *ACTA 2* and *FN* mRNA (Figure S7, Supporting Information) confirming that the proposed method exhibits excellent selectivity. After transfection, the probe experienced a certain degree of degradation due to the cellular environment (Figure S8, Supporting Information), however, it generally maintained a stable level within the 12‐hour period. Given that the amount of probe used was significantly in excess relative to the mRNA content, the effects of the cellular environment and RNA degradation are acceptable under the requirements of our experiment. There were no significant differences in cell viabilities among LX‐2 cells treated with lipofectamine, probes, and the probes in TO1‐Biotin contained buffer (Figure S9, Supporting Information), which indicates that the RNA probes display excellent biocompatibility and minimal cytotoxicity in practical applications. To evaluate the specificity of the fluorescent signals, we designed a Cy3‐labeled fluorescence in situ hybridization (FISH) (Figure 3B) targeting another specific sequence of *COL1A1* mRNA in fixed LX‐2 cells. Cells expressing P1‐1 and P2‐1 exhibited a strong green, fluorescent signal surrounding the nucleus after being incubated with TO1‐Biotin, whereas negligible signals were observed in cells incubated with TO1‐Biotin without transfected probes (Figure 3C; Figure S10, Supporting Information). The intensities of both green (P1‐1 and P2‐1) and red (Cy3‐labeled co‐localization FISH probe) signals increased following TGF‐β stimulation of LX‐2 cells to activate HSCs (Figure 3D). Furthermore, perfect co‐localization of the green and red signals was observed, confirming the identity of the signals as *COL1A1* mRNA (Figure 3E).

Encouraged by the above results, we applied the proposed probes to visualize *COL1A1* mRNA in live LX‐2 cells. An inert Pepper (iPepper)^[^ ^42^ ^]^ with a small fluorophore of HBC620, which emits remarkable red fluorescence only after recognizing another specific sequence (T4, Table S5, Supporting Information) of *COL1A1* mRNA, was introduced for co‐localization with our proposed probes (Figure 3F; Figure S11, Supporting Information). As predicted, the TGF‐β stimulation of LX‐2 cells showed bright green (the proposed probes) and red fluorescent (iPepper with HBC620) foci in the cytoplasm, distinguishing them from the untreated vehicle and cell treated with both TGF‐β and PFD (Figure 3G). The data aligned with the RT‐qPCR results, confirming a positive correlation between the fluorescent signal (Figure 3H) in the confocal images and the levels of *COL1A1* mRNA in live LX‐2 cells (Figure 2F). Co‐localization of green (RNA probes with TO1‐Biotin) and red (iPepper with HBC 620) was observed (Figure 3I), indicating that the bright signals correspond to *COL1A1* mRNA in live cells. To further demonstrate that the cellular signal originates from the *COL1A1* mRNA localized by our fluorescent probe, we utilized the negative cell line HEK‐293T for *COL1A1* mRNA and confirmed its presence through RT‐PCR analysis (Figure S12, Supporting Information). Following the transfection of the probes into these cells, the results indicated that there was no significant fluorescent signal in the cytoplasm of HEK‐293T cells, thereby confirming that the fluorescent signal observed in LX‐2 cells is derived from *COL1A1* mRNA. Interestingly, these probes demonstrated effective visualization of *COL1A1* mRNA in kidney fibroblasts (NRK‐49F), lung fibroblasts (MRC‐5),^[^ ^43^ ^]^ and cardiac fibroblasts cells (CFs),^[^ ^44^ ^]^ indicating its universality in different organ fibrosis (Figures S13–S16, Supporting Information). These findings suggest that the dual‐probe system can serve as a robust fluorescent reporter, facilitating the mapping of *COL1A1* mRNA in both fixed and live cells in various organs.

### 2.4 Screening of Natural Products for Alleviating Liver Fibrosis

**Figure 4:**
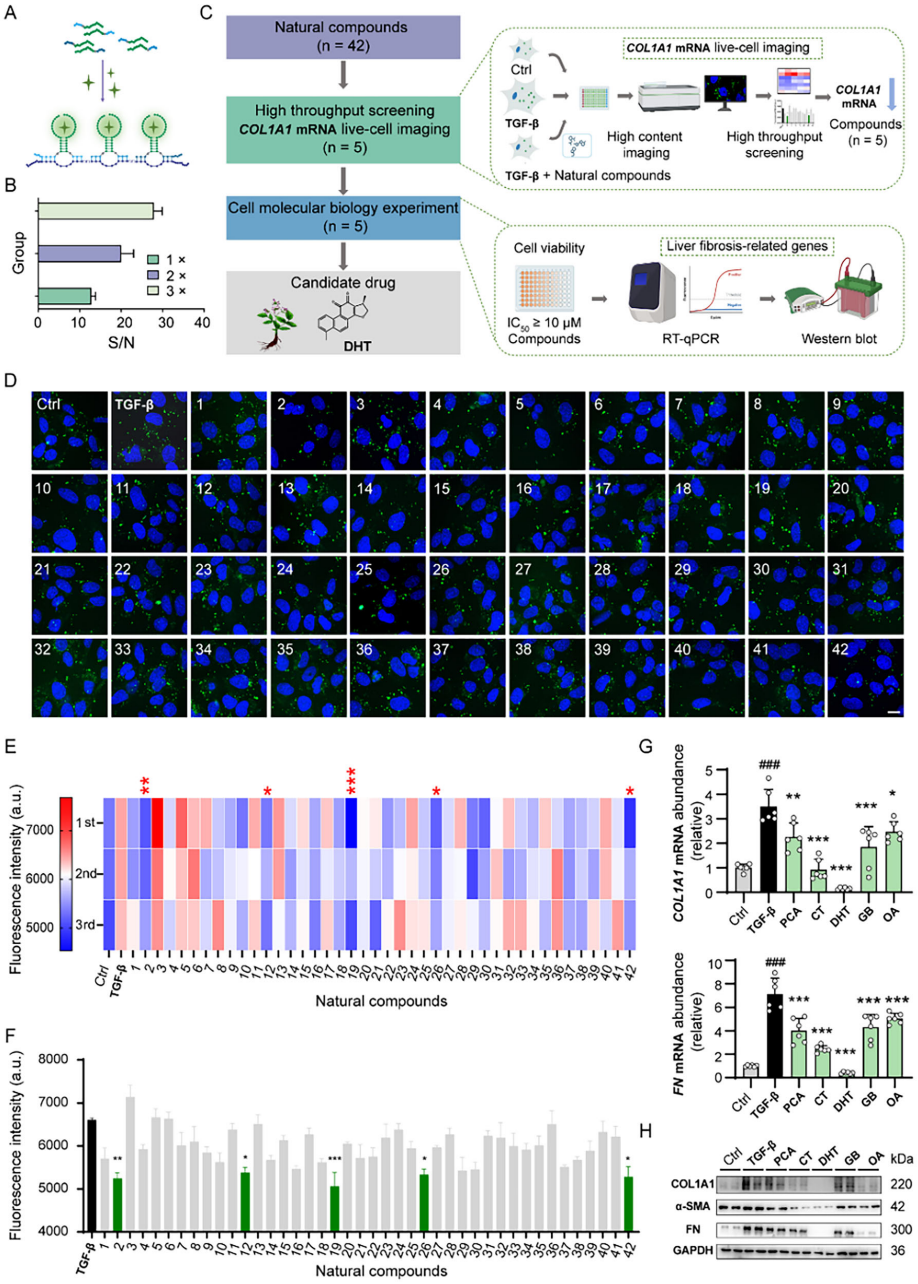
Screening of natural products for alleviating liver fibrosis by high throughput live‐cell screening. Schematic diagram A) and fluorescence intensity B) of the number of probes groups for *COL1A1* mRNA labeling with TO1‐Biotin. C) Schematic representation of the drug screening process. D) LX‐2 cells were stimulated with 10 ng mL^−1^ TGF‐β and treated with 10 µM of various natural compounds (n = 42) for 24 h, respectively. High throughput live‐cell imaging was performed, with green fluorescence indicating *COL1A1* mRNA expression levels. Scale bar = 5 µm. E, F) Heatmap (E) and Bar graph (F) showing fluorescence intensity from high throughput live‐cell imaging. G) mRNAs expression levels of *COL1A1* and *FN* in LX‐2 cells, stimulated with TGF‐β (10 ng mL^−1^, 24 h) and simultaneously treated with or without PCA, CT, DHT, GB and OA (10 µM, 24 h) (n = 6). H) Western blot analysis of LX‐2 cells treated with 10 ng mL^−1^ TGF‐β with or without 10 µM of five leading natural compounds of PCA, CT, DHT, GB, and OA.

Traditional herbal medicines have provided a wealth of knowledge regarding their efficacy and safety through extensive use over time. Building on the promising results from imaging *COL1A1* mRNA in live cells, we have adopted this strategy to identify potent compounds from natural product library that may alleviate liver fibrosis. To achieve superior resolution and brightness, we designed three pairs of probes containing recognition sequences that are complementary to three specific regions within *COL1A1* mRNA (**Figure** **4**A, Table S4, Supporting Information) and testify their fluorescence intensity binding with TO1‐Biotin after target recognition. As evident from Figure 4B, through self‐assembly of probes in vitro, the fluorescent signal corresponding to the three recognition regions was observed to be 2.75 times higher compared to the monomer recognition unit, which is consistent with the imaging results (Figure S17, Supporting Information), thereby facilitating enhanced visualization. In addition, we developed a MBs‐based approach, which was applied in parallel for imaging *COL1A1* mRNA in LX‐2 cells. The proposed probes, along with MBs, can achieve excellent results in imaging *COL1A1* mRNA. Furthermore, by increasing the number of targets recognized by our probes, the brightness and signal‐to‐noise ratio of cellular imaging can be significantly enhanced. The developed probes were then utilized to search potential compounds from natural products library that could alleviate liver fibrosis by monitoring the expression of *COL1A1* mRNA in TGF‐β‐stimulated LX‐2 cells. As described in Figure 4C, natural products screening was first performed for identifying potential compounds that could reduce *COL1A1* mRNA expression in activated LX‐2 cells. Cell viability in normal hepatocytes/HSCs and fibrosis‐related gene changes in HSCs were used to further verify their safety and efficacy. Obviously, the fluorescence imaging results clearly revealed *COL1A1* mRNA expression in the TGF‐β activated LX‐2 cells after being treated with each compound in our natural product library (Figure 4D; Figure S18, Supporting Information). According to the heatmap and quantitative analysis by utilizing software to quantify the imaging intensity, ginkgolide B (GB), oleanolic acid (OA), DHT, protocatechualdehyde (PCA), cryptotanshinone (CT) out of the screened 42 compounds (Table S6, Supporting Information) were identified to show the top 5 inhibitory effects on *COL1A1* expression (Figure 4E,F).

Furthermore, RT‐qPCR (Figure 4G and Table S7, Supporting Information) results showed that both *COL1A1* and *FN* mRNA were significantly downregulated upon treatment with DHT, CT, GB, PCA and OA in LX‐2 cells, which was in accordance with the screening results. Subsequent evaluation using Western blot (WB) analysis (Table S8, Supporting Information) indicated that the five potent natural compounds exhibited a remarkable reduction in the protein levels of FN and COL1A1, as well as α‐SMA in TGF‐β‐stimulated LX‐2 cells, as depicted in Figure 4H, implying their possible anti‐fibrotic effect. Among them, DHT (10 µM), a key active ingredient from *Salvia miltiorrhiza*,^[^ ^38^ ^]^ was identified to show significant activity to reduce *COL1A1* mRNA levels by 95.5%. The cytotoxicity assay (Figures S19 and S20, Supporting Information) demonstrates that DHT exerts a certain degree of toxicity on HSCs at a concentration of 10 µM but does not manifest cytotoxicity toward normal hepatocytes. Therefore, we hypothesize that the mechanism of mechanism of DHT on HSCs involves the downregulation of *COL1A1* mRNA levels, inhibiting HSCs proliferation, which represents a potential therapeutic approach for treating liver fibrosis.

### 2.5 The mRNA Dynamics in Live Cells Respond to Natural Compound Treatment

**Figure 5:**
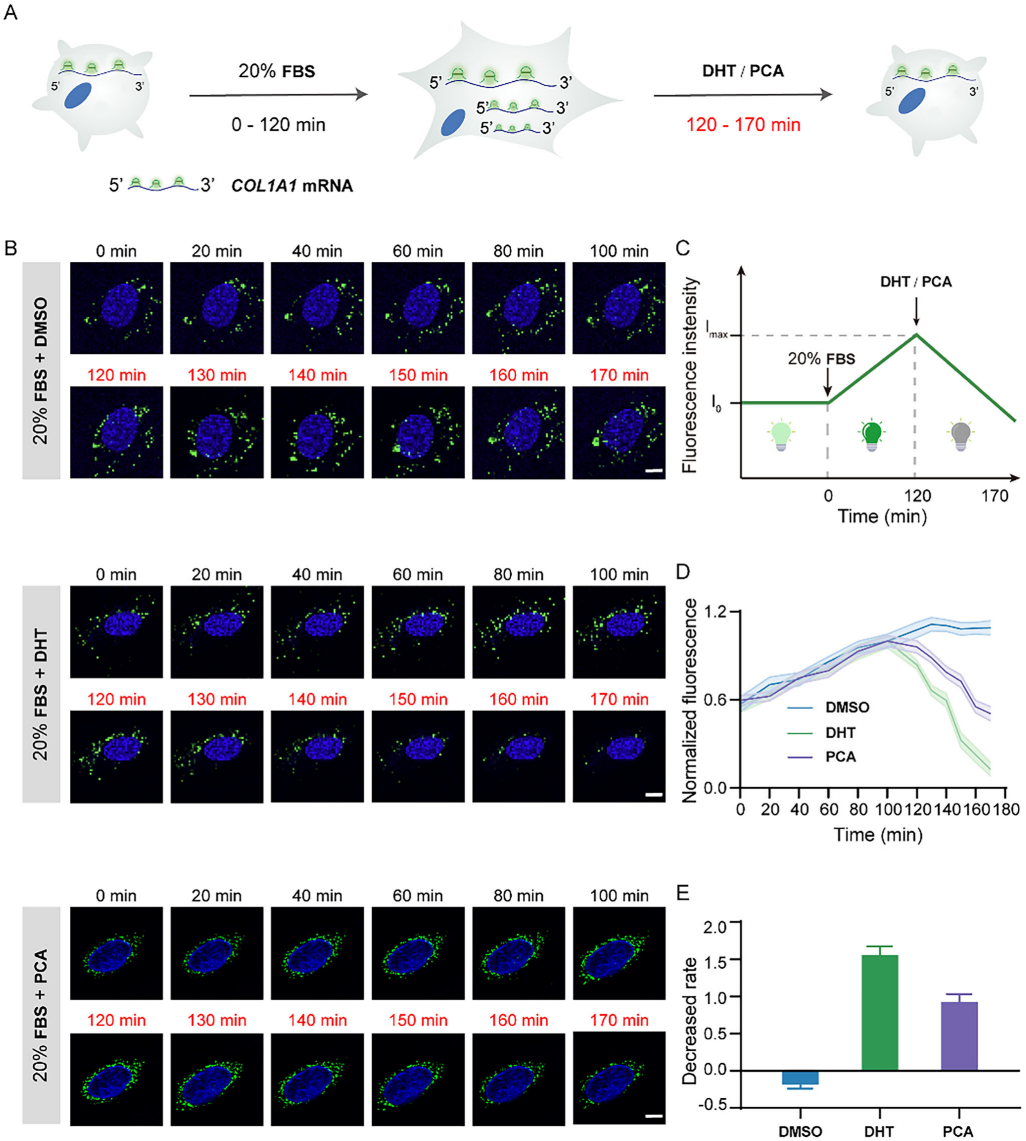
Time‐dependent imaging of *COL1A1* mRNA dynamics in live cells. A) Labeling *COL1A1* mRNA with our probes for live‐cell imaging. LX‐2 cells were treated with 20% FBS for 120 min, followed by the addition of 10 µM DHT or PCA for 30 min. B) Fluorescence images of *COL1A1* mRNA expression in LX‐2 cells treated under different conditions. LX‐2 cells were treated with DMSO, 10 µM DHT, or 10 µM PCA. Green dots represent *COL1A1* mRNA. Scale bar = 5 µm. C) Modelling of *COL1A1* mRNA expression based on our design. D) Fluorescence intensity changes of *COL1A1* mRNA expression of 20% FBS stimulated LX‐2 cells under different compounds treatment. E) The downregulation rate of *COL1A1* mRNA expression under DMSO, DHT, and PCA treatment.

Inspired by the satisfying results of screening compounds for alleviating liver fibrosis, the three pairs of probes were further investigated for spatiotemporal precise visualization of *COL1A1* gene dynamics before and after drug treatment in the 20% FBS‐treated LX‐2 cells. RT‐qPCR results indicated that the *COL1A1* mRNA expression in LX‐2 cells was significantly increased after treating with 20% FBS for 120 min (Figure S21, Supporting Information), thus natural compounds were chosen to add at 120 min (**Figure** **5**A). The Z‐factor, a measure of assay quality, was calculated to be 0.61 according to the separation band (or signal window) between the positive and negative controls relative to their variability, indicating a reliable and reproducible screening system. DHT and PCA have been selected to serve as the leading and general compounds, respectively, for further investigation. As seen from Figure 5B and Video S1 (Supporting Information), bright green fluorescence was detected in LX‐2 cells, suggesting that the three pairs of RNA probes were self‐assembled into RNA Mango II structure induced by endogenous *COL1A1* mRNA. During the application of 20% FBS, the dispersed *COL1A1* mRNA fluorescent signal in individual cells rapidly increased and accumulated, which symbolized the activation of HSCs. Upon treatment of DHT or PCA for 50 min, the bright green fluorescence gradually decreased until it almost disappeared, indicating the effectiveness of DHT and PCA in downregulation of *COL1A1* mRNA. Under the same experimental condition, DMSO‐treated control group remained highly fluorescent, further proving that the changes in the fluorescent signal were caused by natural compounds rather than by photobleaching.

Based on the imaging results, we predict that the trend of cellular fluorescence intensity will initially increase and then decrease, with the rate of decline for DHT being greater than that for PCA (Figure 5C) and then analyzed the fluorescence intensity of images in Figure 5B. In agreement with the hypothesis, the average fluorescence intensities of the green foci increased along with the increasing time in the first 0 to 120 min, and have varying degrees of reduction upon the addition of DHT or PCA, whereas the signal in the control remains high (Figure 5D), further indicating that the fluorescent signal was positively correlated with the level of *COL1A1* mRNA. We further estimated the reduction rate on *COL1A1* mRNA by quantifying the ratio of decreased signal from the probes in individual cells in real time. As seen in Figure 5E, as expected, DHT treatment induced a more significant decrease in the proposed probe signals of *COL1A1* mRNA than that of PCA‐treated cells, while its expression in the control group was still rising, indicating its significant effect in alleviating liver fibrosis. These results demonstrate the ability of our probe to dynamically capture expression changes of mRNA, which hold great promise in clinical drug screening.

### 2.6 In *vivo* Efficacy of Screened Natural Compounds in Alleviating Liver Fibrosis

**Figure 6:**
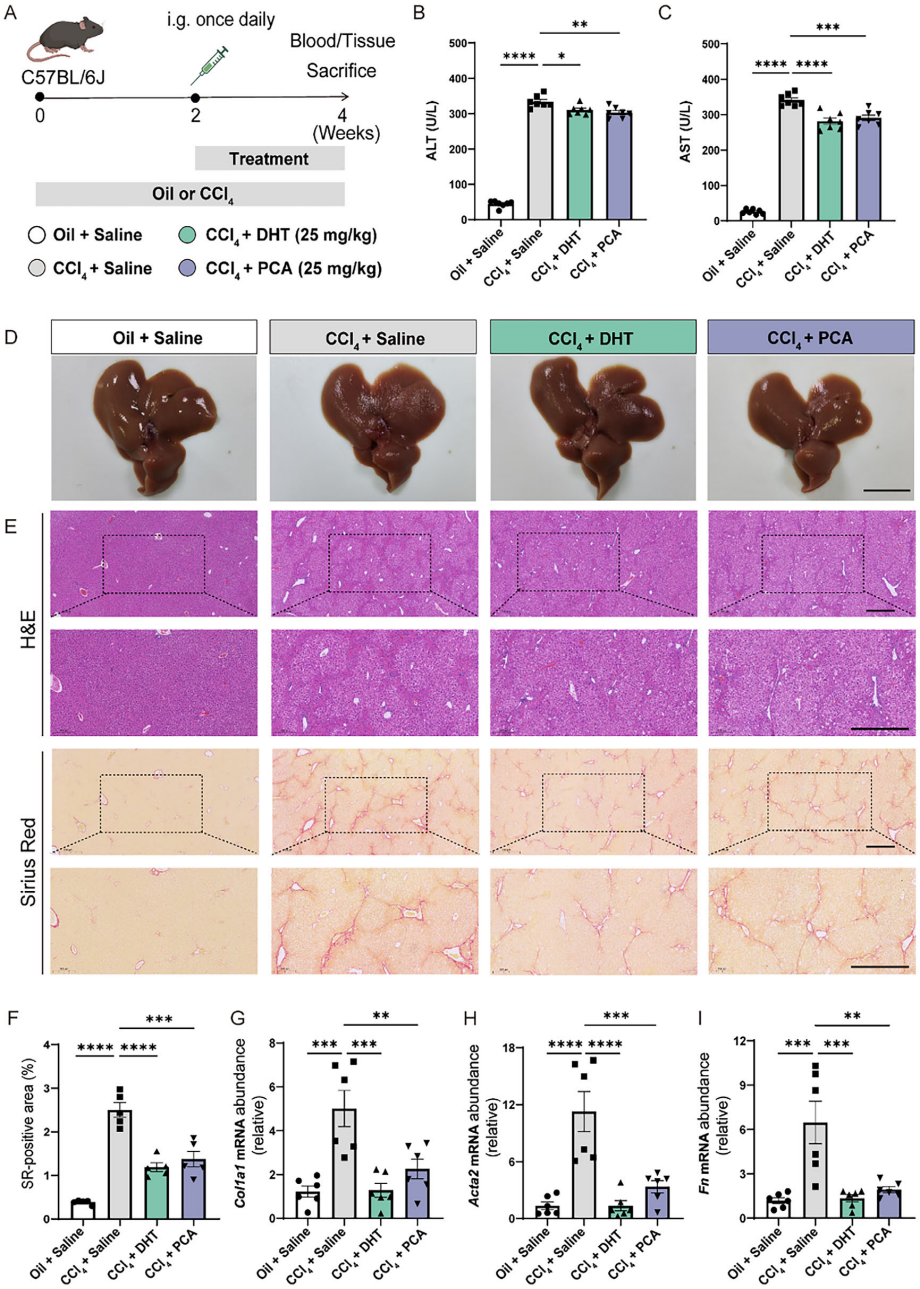
DHT and PCA as potential anti‐liver fibrosis drug candidates. A) Schematic overview of the experimental design, grouping, and drug administration (25 mg kg^−1^ DHT or 25 mg kg^−1^ PCA) in the CCl_4_‐induced model mice. B, C) Serum levels of ALT (B) and AST (C) in CCl_4_‐induced liver fibrosis model group and treatment (DHT or PCA) mice. D, E) Representative images of liver tissues (Scale bar = 1 cm) (D) and stained with H&E and Sirius Red (Scale bar = 500 µm) in CCl_4_‐induced mice after two weeks of drug administration (E). F) Positive area quantification of Sirius Red staining. G–I) mRNAs expression levels of *Col1a1* (G), *Fn* (H), and *Acta2* (I) in the liver of CCl_4_‐induced liver fibrosis model group and treatment (DHT or PCA) group mice (n = 6).

We further explored the in *vivo* therapeutic potential of the screened two natural compounds in liver fibrosis. Carbon tetrachloride (CCl_4_) was selected to induce liver fibrosis in C57BL/6J mice. CCl_4_ (2 µL g^−1^) was injected intra‐peritoneally twice per week into the mice for 4 weeks.^[^ ^11^, ^12^ ^]^ After 2 weeks, the mouse in model groups received DHT (25 mg kg^−1^)^[^ ^45^, ^46^ ^]^ or PCA (25 mg kg^−1^)^[^ ^47^ ^]^ for 2 consecutive weeks (**Figure** **6**A). Serum aminotransferase can provide valuable insights for assessing hepatocellular damage.^[^ ^48^ ^]^ As shown in Figure 6B,C, after 4 weeks of CCl_4_ treatment, both alanine aminotransferase (ALT) and aspartate transaminase (AST) levels in serum of the mouse were significantly increased compared to the oil control group (*p* < 0.0001), indicating liver injury. Obviously, the levels of AST and ALT were reduced in the mouse treated with DHT or PCA, respectively, indicating their positive therapeutic effects on liver injury.

After drug treatment, hepatic fibrosis was markedly attenuated in mice with CCl₄‐induced liver injury (Figure 6D). In the H&E‐stained histological and Sirius Red staining (Figure 6E), the liver tissue in CCl_4_‐treated mice exhibited an excessive stromal vascular presence, accompanied by hemorrhage, inflammatory cell infiltration, and fibrous septa proliferation, with pronounced fibrosis. Conversely, mice treated with DHT or PCA exhibited varying degrees of improvement in liver tissue morphology. Quantitative analysis of the positive area from Sirius Red staining (Figure 6F) further indicated that mice treated with DHT demonstrated more substantial improvements compared to those treated with PCA, suggesting that DHT possesses positive effects against fibrosis. As expected, the upregulations of *Col1a1*, *Fn*, and *Acta2* mRNA in CCl_4_‐induced liver fibrosis mice were suppressed in mouse treated with DHT or PCA (Figure 6G,I). Please take note that the genes under discussion in this context are denoted with only the initial letter in uppercase (*Col1a1*), in contrast to the genes previously addressed, which were presented in uppercase (*COL1A1*). This variation in notation is due to the fact that the mRNA sequences being referenced here are murine origin, whereas the genes discussed earlier are of human origin.^[^ ^49^ ^]^ These results validate the potential of DHT as viable candidates for anti‐liver fibrosis pharmaceuticals, suggesting that this method could be a promising strategy for drug screening aimed at mitigating liver fibrosis to address clinical requirements.

## 3 Conclusion

This study introduces an in‐situ assembly of fluorogenic RNA approach for screening natural anti‐liver fibrosis compounds and dynamic visualization of endogenous *COL1A1* mRNA in live cells. It is designed by inserting an RNA recognition sequence into the optimized split Mango II aptamer, which facilitates the assembly of dual‐RNA probes, leading to the formation of an RNA Mango II structure. This structure significantly enhances the fluorescence of TO1‐Biotin. By tailoring the probes' specific recognition units to target the *COL1A1* mRNA sequence, a biomarker indicative of liver fibrosis. This approach enables noninvasive monitoring of liver fibrosis levels within both fixed and live cells. Utilizing these probes, we screened 42 natural compounds and identified DHT from medicinal herb *Salvia miltiorrhiza*, as the most potent in downregulating *COL1A1* mRNA expression in activated HSCs. Moreover, we successfully monitored the dynamic changes of *COL1A1* mRNA levels in live cells following DHT treatment. The therapeutic potential of the DHT was subsequently validated in *vivo*. The establishment of these probes for drug screening in live cells does not require prior modification of mRNA, and provides a more physiologically relevant evaluation of drug efficacy, circumventing the information loss associated with cell lysis. These probes offer both cost efficiency and universality, enabling the visualization of any position of interest and the level of fibrosis in various organs, such as the lung, kidney, and heart. While the split Mango II aptamer system has proven effective for intracellular RNA imaging in our study, we acknowledge the potential limitations associated with this technology, including non‐specific interactions and inherent background fluorescence as previously documented.^[^ ^50^ ^]^ Importantly, our comprehensive control experiments and quantitative analysis demonstrate that the system maintains an acceptable signal‐to‐noise ratio under our specific experimental conditions. This technical validation confirms that the observed fluorescence signals reliably reflect target RNA dynamics without compromising the core conclusions of this work. It should be noted that emerging RNA imaging systems, such as the Okra‐ACE^[^ ^26^ ^]^ and Clivia‐NBSI^[^ ^51^ ^]^ platforms, have recently demonstrated superior fluorescence intensity and reduced background noise compared to traditional aptamer‐based systems. Although the current Mango II system suffices for the proof‐of‐concept applications described here (mRNA tracking and drug response evaluation), we recognize the potential value of integrating these advanced reporting systems. Such technical upgrades could further enhance imaging resolution and experimental throughput for both basic research and pharmaceutical screening applications. Systematic comparison and optimization of these RNA imaging tools will constitute a key focus of our subsequent investigations. We believe that this study offers a powerful platform for exploring mRNA functionality and drug interactions in various biological events.

## Conflict of Interest

The authors declare no conflict of interest.

## Supporting information



Supporting Information

Supplementary Video S1

## Data Availability

The data that support the findings of this study are available from the corresponding author upon reasonable request.
