# Supplementary material for: In Situ Assembly of Fluorogenic RNA for Screening Natural Anti‐Liver Fibrosis Products via Dynamic Visualization of COL1A1 mRNA
Source: Adv Sci (Weinh). 2025 May 23;12(30):e02850. doi: 10.1002/advs.202502850 (PMC12376682; doi:10.1002/advs.202502850)
Supplement: Supplementary file 1 — Supporting Information [file ADVS-12-e02850-s002.docx]

**Supplementary Information for**

In Situ Assembly of Fluorogenic RNA for Screening Natural Anti-Liver Fibrosis Products via Dynamic Visualization of *COL1A1* mRNA

Rui Bai ^[a], +^, Li-Zeng Zhu ^[b], +^, Changfa Shao ^[a]^, Zheng Yin ^[c]^, Qun Liu ^[b],^ ^*^, Yu Gu ^[a],^ ^*^ and Bin Liu ^[c], *^

1. R. Bai, C. Shao, Y. Gu. School of Materials Science and Engineering, Suzhou University of Science and Technology, Kerui Road, Suzhou 215009, P. R. China. E-mail: guyujcb@163.com
2. L. Zhu, Q. Liu. State Key Laboratory of Natural Medicines, School of Traditional Chinese Pharmacy, China Pharmaceutical University, Nanjing 210009, P. R. China. E-mail: liuquncpu@126.com
3. Z. Yin, B. Liu. Department of Chemical and Biomolecular Engineering, National University of Singapore, Singapore 117585. E-mail: cheliub@nus.edu.sg

[+] These authors contributed equally to this work.

**Content**

[**1.** **Experimental section** **1**](#_Toc195651398)

[**Chemicals** **1**](#_Toc195651399)

[**Apparatus** **1**](#_Toc195651400)

[**Methods** **2**](#_Toc195651401)

[Synthesis of TO1-Biotin 2](#_Toc195651402)

[Spectroscopic measurements 3](#_Toc195651403)

[Gel electrophoresis analysis 3](#_Toc195651404)

[Animals and treatment 3](#_Toc195651405)

[Cell culture and treatment 4](#_Toc195651406)

[Transfection of Cells 4](#_Toc195651407)

[Co-localization 4](#_Toc195651408)

[Live-cell imaging 5](#_Toc195651409)

[High throughput live-cell screening 5](#_Toc195651410)

[Cell Viability Assays 5](#_Toc195651411)

[Quantitative real-time PCR (RT-qPCR) 6](#_Toc195651412)

[Western blot 6](#_Toc195651413)

[H-E stain histological section 6](#_Toc195651414)

[Sirius Red -stained histological section 7](#_Toc195651415)

[Statistics 7](#_Toc195651416)

[**2.** **Sequences and antibodies employed in these work** **7**](#_Toc195651417)

[**Table S1** **Sequences employed in figure 1 A for optimization** **7**](#_Toc195651418)

[**Table S2 Sequences used in the specificity** **8**](#_Toc195651419)

[**Table S3 Sequence of *COL1A1* mRNA** **9**](#_Toc195651420)

[**Table S4 Sequences employed in targeted *COL1A1* mRNA and other mRNA** **12**](#_Toc195651421)

[**Table S5 Sequences for colocalization experiment** **12**](#_Toc195651422)

[**Table S6 Name of natural compounds in high throughput screening experiment** **12**](#_Toc195651423)

[**Table S7 Primers for RT-qPCR** **13**](#_Toc195651424)

[**Table S8 Antibodies for immunoblotting** **13**](#_Toc195651425)

[**3.** **Figures** **14**](#_Toc195651426)

[**Figure S1** **14**](#_Toc195651427)

[**Figure S2** **14**](#_Toc195651428)

[**Figure S3** **15**](#_Toc195651429)

[**Figure S4** **15**](#_Toc195651430)

[**Figure S5** **16**](#_Toc195651431)

[**Figure S6** **16**](#_Toc195651432)

[**Figure S7** **17**](#_Toc195651433)

[**Figure S8** **17**](#_Toc195651434)

[**Figure S9** **18**](#_Toc195651435)

[**Figure S10** **18**](#_Toc195651436)

[**Figure S11** **19**](#_Toc195651437)

[**Figure S12** **21**](#_Toc195651438)

[**Figure S13** **20**](#_Toc195651439)

[**Figure S14** **21**](#_Toc195651440)

[**Figure S15** **22**](#_Toc195651441)

[**Figure S16** **23**](#_Toc195651442)

[**Figure S17** **24**](#_Toc195651443)

[**Figure S18** **24**](#_Toc195651444)

[**Figure S19** **25**](#_Toc195651445)

[**Figure S20** **26**](#_Toc195651446)

[**Figure S21** **26**](#_Toc195651447)

1. **Experimental section**

**Chemicals**

Protocatechuic aldehyde was sourced from Sigma-Aldrich. Dihydrotanshinone I was obtained from Jiangsu Yong Jian Pharmaceuticals. ALT and AST assay kits were acquired from Nanjing Jian Cheng Bioengineering Institute (Nanjing, China). HBC620 was procured from Med Chem Express (Shanghai, China). Hoechst 33342 was purchased from Sigma-Aldrich. Lipofectamine 3000 was supplied by Thermo Fisher Scientific Inc. Fetal bovine serum was procured from New Zerum. Cell Counting Kit-8 (CCK-8), qPCR SYBR Green Master Mix, and cDNA synthesis kit were obtained from Yeasen Biotechnology (Wuhan, China). RNA extraction reagent was sourced from Vazyme Biotech (Nanjing, China). High-glucose Dulbecco's Modified Eagle Medium (DMEM) and Roswell Park Memorial Institute (RPMI) 1640 media were purchased from Jiangsu Keygen Biotech. Diethyl pyrocarbonate (DEPC)-treated water was acquired from Tiangen Biotech. All sequences used in this study were synthesized by Gen Script Biotech (Nanjing, China), and were of analytical grade.

**Apparatus**

Fluorescent spectra were measured with a Shimadzu RF-6000 fluorescence spectrophotometer. Cell imaging was conducted with a TCS SP8 laser confocal microscope. High throughput live-cell imaging for drug screening was performed using the Opera Phenix system from PerkinElmer. The Quant Studio 3 real-time PCR system by Thermo Fisher Scientific was employed for real-time quantitative PCR analyses. High-sensitivity structured illumination microscopy (HIS-SIM) was conducted using equipment provided by CSR Biotech (Guangzhou, China).

**Methods**

**Synthesis of TO1-Biotin**

We synthesized the small-molecule fluorophore TO1-Biotin, which binds to the Mango II aptamer, using established methodologies described in the literature^[1]^.

1. NMR Spectrum of TO1-Biotin

For the compound TO1-Biotin, the ^1^H NMR (400 MHz, D_2_O) δ 8.54 (d, *J* = 8.4 Hz, 1H), 8.45 (d, *J* = 8.4 Hz, 1H), 8.05 – 7.99 (m, 2H),7.87 (d, *J* = 8.5 Hz, 1H), 7.79 (t, *J* = 8.5 Hz, 1H), 7.58 – 7.51 (m, 2H), 7.49 (t, *J* = 8.5 Hz, 1H), 7.38 (d, *J* = 8.4 Hz, 1H), 6.83 (s, 1H), 5.27 (s, 2H), 4.44 (d, *J* = 12.3 Hz, 1H), 4.23 (dd, *J* = 12.4, 4.4 Hz, 1H), 4.21 (s, 3H), 3.64 − 3.58 (m, 11H), 3.55 − 3.47 (m, 5H), 3.19 − 3.10 (m, 1H), 2.86 (dd, *J* = 12.8, 4.4 Hz, 1H), 2.66 (d, *J* = 12.8 Hz, 1H), 2.14 (t, *J* = 7.2 Hz, 2H), 1.75 − 1.40 (m, 4H), 1.39 − 1.27 (m, 2H).

**
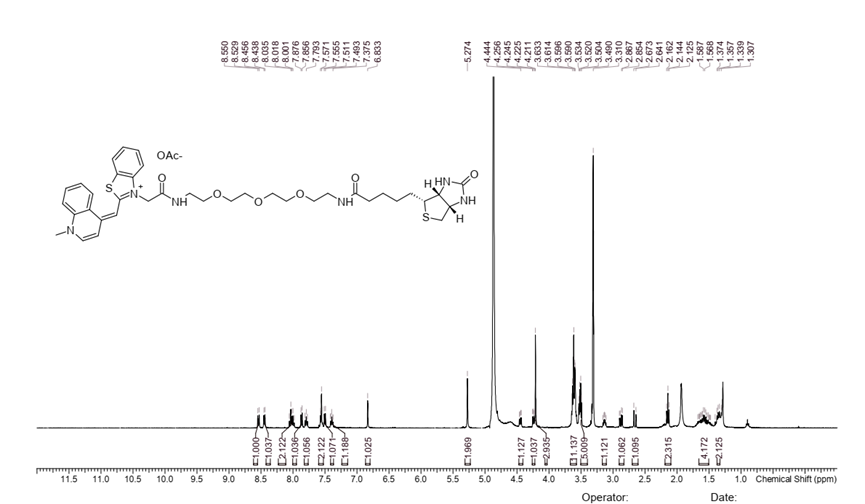
**

^1^H NMR spectrum of TO1-Biotin in D_2_O

1. MS Spectra of TO1-Biotin

For the compound TO1-Biotin, the exact mass calculated for [M+H^+^, C_38_H_49_N_6_O_6_S_2_^+^] has a calculated *m/z* of 749.3. The observed *m/z* is 749.4.
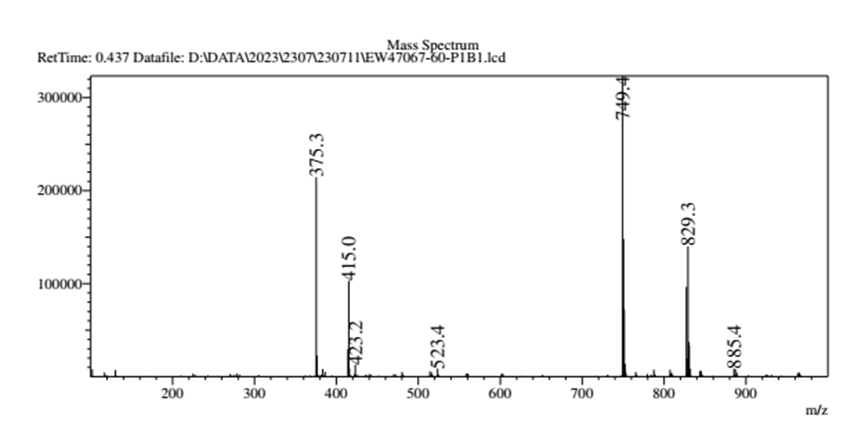


MS Spectrum of TO1-Biotin in Positive Ion Mode

**Spectroscopic measurements**

All DNA strands and RNA were stored in a HEPES buffer (40 mM, pH 7.4) containing 10 µM TO1-Biotin, 100 mM KCl, and 5 mM MgCl_2_. This buffer was prepared using DEPC-treated water and samples were kept at -20℃. Prior to use, all samples were heated at 95℃ for 5 min and then allowed to gradually cool to room temperature. Reagents were prewarmed at 37℃ for 30 min before mixing. After incubation at 37℃ for 2 h, 10 µM TO1-Biotin was added and the mixture was incubated for an additional 30 min. Subsequently, fluorescence emission spectra were measured using a Macro Cuvette with an excitation wavelength of 505 nm and a slit width of 5 nm.

**Gel electrophoresis analysis**

For the gel electrophoresis experiment, 6 μL of a mixed solution (prepared at a 5:1 volume ratio of sample to loading buffer) was loaded onto a 10% polyacrylamide gel. Electrophoresis was conducted at 80 V for 120 min in 1× TBE buffer (comprising 50 mM Tris, 50 mM HEPES, and 2 mM EDTA, pH 7.9). Subsequently, the gel was scanned using a gel imaging analysis system.

**Animals and treatment**

All animal experiments were approved by the Animal Care and the Animal Ethics Committee of China Pharmaceutical University (protocol no.2024-09-117). C57BL/6J mice (8-week-old, male) were purchased from Jiangsu Gempharmatech Biotechnology (Nanjing, China), and maintained in a SPF class environment with a 12 h light-dark cycle at 24-26℃ and free access to food and water. Mice were fed a normal chow diet (10% kcal from fat; Xietong Organism, China). To establish the CCl_4_-induced liver fibrosis model, mice were injected CCl_4_ (Aladdin, China) at the dose of 0.2 μL g ^-1^of body weight intra-peritoneally twice per week. Mice were treated with DHT (25 mg kg^-1^) or PCA (25 mg kg^-1^) once daily via oral gavage for the last two weeks.

**Cell culture and treatment**

The human HSC line LX-2 was cultured in RPMI-1640 (KeyGEN Biotech, KGL1501-500, China) with 10% fetal bovine serum (FBS) and 1% penicillin/streptomycin (P/S, Invitrogen, Carlsbad, CA, USA). NRK-49F, MRC-5 and Cardiac fibroblasts were cultured in DMEM (KeyGEN Biotech, KGL1501-500, China) with 10% FBS and 1% penicillin/streptomycin. All of these cells were grown in a 5% CO_2_ humidified atmosphere at 37℃. LX-2 cells were activated by 10 ng mL^-1^ TGF-β for 24 h or 20% FBS for 2-12 h after seeding in the plate.

**Transfection of cells**

LX-2 cells were transiently transfected with the probe using Lipofectamine® 3000 transfection reagent (ThermoFisher, America) according to the manufacturer’s instructions. 200 ng probe or 1 μg ipepper was combined with 5 μL of Lipofectamine® 3000 reagent and 100 μL Opti-MEM (Gibco, America). The complexes were incubated at room temperature for 20 min to prepare probe-lipid complexes. LX-2 cells were plated into a 20 mm dish (Cellvis, America) and grown to ∼80% confluent at the time of transfection. When transfection, original medium of LX-2 cells was changed to 400 μL OPTI-MEM and added 100 μL probe-lipid complexes, and then incubated at a 37℃ 5% CO_2_ environment for 4 h. After 4 h, Culture medium was changed to RPMI-1640 with 10% FBS and 1% penicillin/streptomycin.

**Co-localization**

For live-cell co-localization, LX-2 cells were transfected with probe or ipepper for 4 h, incubated with TO1-Biotin or HBC620 for 40 min, and then incubated with Hoechst for 10 min. For fixed-cell co-localization, LX-2 cells were transfected with the probes and fixed with 4% paraformaldehyde at RT for 15 min, permeabilized and then incubated with TO1-biotin and Cy3-labeled FISH at 37℃. After incubation with DAPI solution for 10 min, the cells were analyzed using a confocal scanning microscope (Olympus, Japan).

**Live-cell imaging**

LX-2 cells were transiently transfected with the probes for 4 h, incubated with TO1-biotin for 40 min, and then incubated with Hoechst for 10 min. After adding 20% FBS to activate LX-2 cells, the sample was imaged with a fluorescence microscope (Leica SP8) equipped with online CO_2_ culture equipment (INUB-WELSX-SET) and 63X objective. The TO1-biotin was imaged using 488 nm laser. Super-resolution imaging using High Sensitivity Structured Illumination (HIS-SIM) (CSR Biotech, China) and 100X objective.

**High throughput live-cell screening**

LX-2 cells were seeded in 96 - well plates at a density of 1×10^4^ cells per well. After the cells adhered overnight, they were treated with 42 different natural compounds (10 μM) for 24 h. Cells treated with TGF-β were used as the positive control, and PBS was used as the negative control. Next, LX-2 cells were transfected with 200 ng probes for 4 h and incubation with TO1-Biotin for 40 min. The cells were then washed with PBS, stained with a hoechst 33342 for 10 min. Subsequently, the cells were imaged using a high-content imaging system (PerkinElmer, America). Cellular fluorescence was measured using high-content imaging, with signal intensity normalized to background and analyzed using automated software to ensure consistency. For each condition, fluorescence signals were quantified from approximately three random 250 μm × 250 μm fields of view to ensure statistical robustness. The total fluorescence intensity within a unit field of view was measured, and the number of cells was counted using Hoechst 33342. The average fluorescence intensity was calculated as the total fluorescence intensity divided by the number of cells. The experiment was repeated three times.

**Cell viability assays**

96 - well plates were used to seed all cells at a density of 1×10^4^ cells per well. The cells were allowed to adhere for 12 h. After that, the original medium was removed. Cells were transfected with probes using Lipofectamine 3000 according to the manufacturer’s instructions. After 4 h, the cells were incubated TO1-Biotin at different concentrations for 40 min. Next, each well was rinsed with PBS and the medium was replaced with CCK-8 medium diluted 1:10 CCK-8 reagent in DMEM at 37℃ for 2 h. Cell viability was measured at a wavelength of 450 nm using a Envision microplate reader (PerkinElmer, America). For the experiment shown in Figure S18, cell viability assays were conducted after cells were treated with 10 μM of different compounds for 24 h.

**Quantitative real-time PCR (RT-qPCR)**

The total RNA was extracted using TRIzol^TM^ Reagent (Vazyme, China) according to the manufacturer’s instructions. Generally, cDNA was synthesized using a Synthesis kit (Yeasen, China). All RT-qPCR reactions were performed on the [QuantStudio 3 Real-Time PCR 96 System](https://www.thermofisher.cn/cn/zh/home/life-science/pcr/real-time-pcr/real-time-pcr-instruments/quantstudio-3-5-real-time-pcr-system/quantstudio-3.html) (ThermoFisher, America) using the SYBR Green Master Mix (Yeasen, China). All genes are normalized to *β-actin* as the endogenous control. All the primer sequences are shown in Supplementary Table S6.

**Western blot**

Liver tissue samples and cultured LX-2 were homogenized in RIPA buffer (Beyotime, China) with 1 mM phenylmethylsulfonyl fluoride (PMSF) (Beyotime, China) on ice. The total protein concentration was quantified with a BCA assay kit (Beyotime, China) for the normalization of assayed samples. Proteins were electrophoresed on 8% SDS-PAGE gels and transferred to PVDF membranes. The membranes were blocked by 5% nonfat milk at RT for 2 h and then incubated with primary antibody (Supplementary Table S7) overnight at 4℃, followed by incubation with the appropriate secondary antibodies coupled to horseradish peroxidase. Proteins were detected by ECL chemiluminescence (Tanon, China).

H&E stain histological section

Mouse liver tissue samples were fixed in 10% formalin for 24 h and rinsed with water. The sections were then dehydrated and cleared through two xylene baths (10 min each) and graded ethanol solutions (absolute ethanol twice, followed by 95%, 90%, 80%, and 70% ethanol), with each step lasting 5 min, followed by another rinse with water. The sections were stained with hematoxylin for nuclei and eosin for cytoplasmic structures. After staining, they were dehydrated again through two rounds of 95% ethanol, absolute ethanol, and xylene, each lasting 5 min, then air-dried, mounted with neutral resin, and examined microscopically.

Sirius red -stained histological section

The Sirius Red staining procedure involves deparaffinizing and rehydrating the tissue samples with xylene and graded alcohols. The sections are stained for one hour at room temperature with a saturated solution of Sirius Red in picric acid, rinsed with acidified water to remove excess dye, and enhance collagen binding specificity. The slides are dehydrated with graded alcohols, cleared with xylene, and mounted. Under polarized light microscopy, Sirius Red-stained collagen fibers show birefringence, appearing bright red or yellow against a dark background, allowing for qualitative and quantitative analysis of collagen deposition.

**Statistics**

All results were presented as mean ± standard error of the mean (SEM) and performed as described in the corresponding figure legends. Comparisons between two groups were analyzed using a two-tailed Student’s *t* test, and those among three or more groups by using one-way analysis of variance (ANOVA). The *p* < 0.05 was considered statistically significant. Statistical significance analyses were performed using GraphPad Prism version 9.0 (GraphPad Software, La Jolla, CA, America).

1. **Sequences and antibodies employed in these work**

**Table S1** **Sequences employed in figure 1 A for optimization**

| **Group** | **No.** | **Sequence 5’-3’** |
| --- | --- | --- |
| ① | 18, 19 | P1: GCA CGU ACG AAG GAG AGG  P2: AGA GGA AGA GGA GAG UAC GUG C |
|  | 19, 20 | P1: GCA CGU ACG AAG GAG AGG A  P2: GA GGA AGA GGA GAG UAC GUG C |
|  | 20, 21 | P1: GCA CGU ACG AAG GAG AGG AG  P2: A GGA AGA GGA GAG UAC GUG C |
|  | 21, 22 | P1: GCA CGU ACG AAG GAG AGG AGA  P2: GGA AGA GGA GAG UAC GUG C |
|  | 22, 23 | P1: GCA CGU ACG AAG GAG AGG AGA G  P2: GA AGA GGA GAG UAC GUG C |
|  | 23, 24 | P1: GCA CGU ACG AAG GAG AGG AGA GG  P2: A AGA GGA GAG UAC GUG C |
| ② | Mango Ⅱ | GCA CGU ACG AAG GAG AGG AGA GGA AGA GGA GAG UAC GUG C |
|  | 1 | P1: CA CGU ACG AAG GAG AGG AGA  P2: GGA AGA GGA GAG UAC GUG |
|  | 2 | P1: A CGU ACG AAG GAG AGG AGA  P2: GGA AGA GGA GAG UAC GU |
|  | 3 | P1: CGU ACG AAG GAG AGG AGA  P2: GGA AGA GGA GAG UAC G |
|  | 4 | P1: GU ACG AAG GAG AGG AGA  P2: GGA AGA GGA GAG UAC |
|  | 5 | P1: U ACG AAG GAG AGG AGA  P2: GGA AGA GGA GAG UA |
|  | 6 | P1:GA CGU ACG AAG GAG AGG AGA  P2: GGA AGA GGA GAG UAC GU C |
|  | 7 | P1: G CGU ACG AAG GAG AGG AGA  P2: GGA AGA GGA GAG UAC G C |
|  | 8 | P1: G GU ACG AAG GAG AGG AGA  P2: GGA AGA GGA GAG UAC C |
|  | 9 | P1: G U ACG AAG GAG AGG AGA  P2: GGA AGA GGA GAG UA C |
| ③ | T | UAG CUU AUC AGA CUG AUG UUG A |
|  | 1 | P1:CA ACA UCA A G CGU ACG AAG GAG AGG AGA  P2: GGU AGA GGA GAG UAC GCA GAU AAG CUA |
|  | 2 | P1: CA ACA UCA A U CGU ACG AAG GAG AGG AGA  P2: GGU AGA GGA GAG UAC GCA GAU AAG CUA |
|  | 3 | P1: CA ACA UCA A A CGU ACG AAG GAG AGG AGA  P2: GGU AGA GGA GAG UAC GCA GAU AAG CUA |
|  | 4 | P1:CA ACA TCA A G CGT ACG AAG GAG AGG AGA  P2: GGU AGA GGA GAG UAC GGA GAU AAG CUA |
|  | 5 | P1:CA ACA UCA A CGU ACG AAG GAG AGG AGA  P2: GGU AGA GGA GAG UAC GA GAU AAG CUA |
|  | 6 | P1:CA ACA UCA A GU ACG AAG GAG AGG AGA  P2: GGU AGA GGA GAG UAC A GAU AAG CUA |
|  | 7 | P1: CA ACA UCA A U ACG AAG GAG AGG AGA  P2: GGU AGA GGA GAG UA A GAU AAG CUA |

Sequence marked in red: The optimal sequence from each group.

**Table S2 Sequences used in the specificity**

| **Name** | **Sequence 5’-3’** |
| --- | --- |
| Target | UAG CUU AUC AGAC UGA UGU UGA |
| P1 | CAA CAU CAA ACG UAC GAA GGA GAGGA A |
| P2 | GGU AGA GGA GAG UAC GCA GAU AAG CUA |
| Mismatch-1 (M1) | UAG CUU AUG AGAC UGA UGU UGA |
| Mismatch-2-1 (M2-1) | UAG CUU UUG AGAC UGA UGU UGA |
| Mismatch-2-2 (M2-2) | UAG CUU AUG AGAC AGA UGU UGA |

Sequence marked in red: The mismatch bases from each group.

**Table S3 Sequence of *COL1A1* mRNA**

| GCAGACGGGAGTTTCTCCTCGGGGTCGGAGCAGGAGGCACGCGGAGTGTGAGGCCACGCATGAGCGGACGCTAACCCCCTCCCCAGCCACAAAGAGTCTACATGTCTAGGGTCTAGACATGTTCAGCTTTGTGGACCTCCGGCTCCTGCTCCTCTTAGCGGCCACCGCCCTCCTGACGCACGGCCAAGAGGAAGGCCAAGTCGAGGGCCAAGACGAAGACATCCCACCAATCACCTGCGTACAGAACGGCCTCAGGTACCATGACCGAGACGTGTGGAAACCCGAGCCCTGCCGGATCTGCGTCTGCGACAACGGCAAGGTGTTGTGCGATGACGTGATCTGTGACGAGACCAAGAACTGCCCCGGCGCCGAAGTCCCCGAGGGCGAGTGCTGTCCCGTCTGCCCCGACGGCTCAGAGTCACCCACCGACCAAGAAACCACCGGCGTCGAGGGACCCAAGGGAGACACTGGCCCCCGAGGCCCAAGGGGACCCGCAGGCCCCCCTGGCCGAGATGGCATCCCTGGACAGCCTGGACTTCCCGGACCCCCCGGACCCCCCGGACCTCCCGGACCCCCTGGCCTCGGAGGAAACTTTGCTCCCCAGCTGTCTTATGGCTATGATGAGAAATCAACCGGAGGAATTTCCGTGCCTGGCCCCATGGGTCCCTCTGGTCCTCGTGGTCTCCCTGGCCCCCCTGGTGCACCTGGTCCCCAAGGCTTCCAAGGTCCCCCTGGTGAGCCTGGCGAGCCTGGAGCTTCAGGTCCCATGGGTCCCCGAGGTCCCCCAGGTCCCCCTGGAAAGAATGGAGATGATGGGGAAGCTGGAAAACCTGGTCGTCCTGGTGAGCGTGGGCCTCCTGGGCCTCAGGGTGCTCGAGGATTGCCCGGAACAGCTGGCCTCCCTGGAATGAAGGGACACAGAGGTTTCAGTGGTTTGGATGGTGCCAAGGGAGATGCTGGTCCTGCTGGTCCTAAGGGTGAGCCTGGCAGCCCTGGTGAAAATGGAGCTCCTGGTCAGATGGGCCCCCGTGGCCTGCCTGGTGAGAGAGGTCGCCCTGGAGCCCCTGGCCCTGCTGGTGCTCGTGGAAATGATGGTGCTACTGGTGCTGCCGGGCCCCCTGGTCCCACCGGCCCCGCTGGTCCTCCTGGCTTCCCTGGTGCTGTTGGTGCTAAGGGTGAAGCTGGTCCCCAAGGGCCCCGAGGCTCTGAAGGTCCCCAGGGTGTGCGTGGTGAGCCTGGCCCCCCTGGCCCTGCTGGTGCTGCTGGCCCTGCTGGAAACCCTGGTGCTGATGGACAGCCTGGTGCTAAAGGTGCCAATGGTGCTCCTGGTATTGCTGGTGCTCCTGGCTTCCCTGGTGCCCGAGGCCCCTCTGGACCCCAGGGCCCCGGCGGCCCTCCTGGTCCCAAGGGTAACAGCGGTGAACCTGGTGCTCCTGGCAGCAAAGGAGACACTGGTGCTAAGGGAGAGCCTGGCCCTGTTGGTGTTCAAGGACCCCCTGGCCCTGCTGGAGAGGAAGGAAAGCGAGGAGCTCGAGGTGAACCCGGACCCACTGGCCTGCCCGGACCCCCTGGCGAGCGTGGTGGACCTGGTAGCCGTGGTTTCCCTGGCGCAGATGGTGTTGCTGGTCCCAAGGGTCCCGCTGGTGAACGTGGTTCTCCTGGCCCTGCTGGCCCCAAAGGATCTCCTGGTGAAGCTGGTCGTCCCGGTGAAGCTGGTCTGCCTGGTGCCAAGGGTCTGACTGGAAGCCCTGGCAGCCCTGGTCCTGATGGCAAAACTGGCCCCCCTGGTCCCGCCGGTCAAGATGGTCGCCCCGGACCCCCAGGCCCACCTGGTGCCCGTGGTCAGGCTGGTGTGATGGGATTCCCTGGACCTAAAGGTGCTGCTGGAGAGCCCGGCAAGGCTGGAGAGCGAGGTGTTCCCGGACCCCCTGGCGCTGTCGGTCCTGCTGGCAAAGATGGAGAGGCTGGAGCTCAGGGACCCCCTGGCCCTGCTGGTCCCGCTGGCGAGAGAGGTGAACAAGGCCCTGCTGGCTCCCCCGGATTCCAGGGTCTCCCTGGTCCTGCTGGTCCTCCAGGTGAAGCAGGCAAACCTGGTGAACAGGGTGTTCCTGGAGACCTTGGCGCCCCTGGCCCCTCTGGAGCAAGAGGCGAGAGAGGTTTCCCTGGCGAGCGTGGTGTGCAAGGTCCCCCTGGTCCTGCTGGTCCCCGAGGGGCCAACGGTGCTCCCGGCAACGATGGTGCTAAGGGTGATGCTGGTGCCCCTGGAGCTCCCGGTAGCCAGGGCGCCCCTGGCCTTCAGGGAATGCCTGGTGAACGTGGTGCAGCTGGTCTTCCAGGGCCTAAGGGTGACAGAGGTGATGCTGGTCCCAAAGGTGCTGATGGCTCTCCTGGCAAAGATGGCGTCCGTGGTCTGACTGGCCCCATTGGTCCTCCTGGCCCTGCTGGTGCCCCTGGTGACAAGGGTGAAAGTGGTCCCAGCGGCCCTGCTGGTCCCACTGGAGCTCGTGGTGCCCCCGGAGACCGTGGTGAGCCTGGTCCCCCCGGCCCTGCTGGCTTTGCTGGCCCCCCTGGTGCTGACGGCCAACCTGGTGCTAAAGGCGAACCTGGTGATGCTGGTGCTAAAGGCGATGCTGGTCCCCCTGGCCCTGCCGGACCCGCTGGACCCCCTGGCCCCATTGGTAATGTTGGTGCTCCTGGAGCCAAAGGTGCTCGCGGCAGCGCTGGTCCCCCTGGTGCTACTGGTTTCCCTGGTGCTGCTGGCCGAGTCGGTCCTCCTGGCCCCTCTGGAAATGCTGGACCCCCTGGCCCTCCTGGTCCTGCTGGCAAAGAAGGCGGCAAAGGTCCCCGTGGTGAGACTGGCCCTGCTGGACGTCCTGGTGAAGTTGGTCCCCCTGGTCCCCCTGGCCCTGCTGGCGAGAAAGGATCCCCTGGTGCTGATGGTCCTGCTGGTGCTCCTGGTACTCCCGGGCCTCAAGGTATTGCTGGACAGCGTGGTGTGGTCGGCCTGCCTGGTCAGAGAGGAGAGAGAGGCTTCCCTGGTCTTCCTGGCCCCTCTGGTGAACCTGGCAAACAAGGTCCCTCTGGAGCAAGTGGTGAACGTGGTCCCCCTGGTCCCATGGGCCCCCCTGGATTGGCTGGACCCCCTGGTGAATCTGGACGTGAGGGGGCTCCTGGTGCCGAAGGTTCCCCTGGACGAGACGGTTCTCCTGGCGCCAAGGGTGACCGTGGTGAGACCGGCCCCGCTGGACCCCCTGGTGCTCCTGGTGCTCCTGGTGCCCCTGGCCCCGTTGGCCCTGCTGGCAAGAGTGGTGATCGTGGTGAGACTGGTCCTGCTGGTCCCGCCGGTCCTGTCGGCCCTGTTGGCGCCCGTGGCCCCGCCGGACCCCAAGGCCCCCGTGGTGACAAGGGTGAGACAGGCGAACAGGGCGACAGAGGCATAAAGGGTCACCGTGGCTTCTCTGGCCTCCAGGGTCCCCCTGGCCCTCCTGGCTCTCCTGGTGAACAAGGTCCCTCTGGAGCCTCTGGTCCTGCTGGTCCCCGAGGTCCCCCTGGCTCTGCTGGTGCTCCTGGCAAAGATGGACTCAACGGTCTCCCTGGCCCCATTGGGCCCCCTGGTCCTCGCGGTCGCACTGGTGATGCTGGTCCTGTTGGTCCCCCCGGCCCTCCTGGACCTCCTGGTCCCCCTGGTCCTCCCAGCGCTGGTTTCGACTTCAGCTTCCTGCCCCAGCCACCTCAAGAGAAGGCTCACGATGGTGGCCGCTACTACCGGGCTGATGATGCCAATGTGGTTCGTGACCGTGACCTCGAGGTGGACACCACCCTCAAGAGCCTGAGCCAGCAGATCGAGAACATCCGGAGCCCAGAGGGCAGCCGCAAGAACCCCGCCCGCACCTGCCGTGACCTCAAGATGTGCCACTCTGACTGGAAGAGTGGAGAGTACTGGATTGACCCCAACCAAGGCTGCAACCTGGATGCCATCAAAGTCTTCTGCAACATGGAGACTGGTGAGACCTGCGTGTACCCCACTCAGCCCAGTGTGGCCCAGAAGAACTGGTACATCAGCAAGAACCCCAAGGACAAGAGGCATGTCTGGTTCGGCGAGAGCATGACCGATGGATTCCAGTTCGAGTATGGCGGCCAGGGCTCCGACCCTGCCGATGTGGCCATCCAGCTGACCTTCCTGCGCCTGATGTCCACCGAGGCCTCCCAGAACATCACCTACCACTGCAAGAACAGCGTGGCCTACATGGACCAGCAGACTGGCAACCTCAAGAAGGCCCTGCTCCTCCAGGGCTCCAACGAGATCGAGATCCGCGCCGAGGGCAACAGCCGCTTCACCTACAGCGTCACTGTCGATGGCTGCACGAGTCACACCGGAGCCTGGGGCAAGACAGTGATTGAATACAAAACCACCAAGACCTCCCGCCTGCCCATCATCGATGTGGCCCCCTTGGACGTTGGTGCCCCAGACCAGGAATTCGGCTTCGACGTTGGCCCTGTCTGCTTCCTGTAAACTCCCTCCATCCCAACCTGGCTCCCTCCCACCCAACCAACTTTCCCCCCAACCCGGAAACAGACAAGCAACCCAAACTGAACCCCCTCAAAAGCCAAAAAATGGGAGACAATTTCACATGGACTTTGGAAAATATTTTTTTCCTTTGCATTCATCTCTCAAACTTAGTTTTTATCTTTGACCAACCGAACATGACCAAAAACCAAAAGTGCATTCAACCTTACCAAAAAAAAAAAAAAAAAAAGAATAAATAAATAACTTTTTAAAAAAGGAAGCTTGGTCCACTTGCTTGAAGACCCATGCGGGGGTAAGTCCCTTTCTGCCCGTTGGGCTTATGAAACCCCAATGCTGCCCTTTCTGCTCCTTTCTCCACACCCCCCTTGGGGCCTCCCCTCCACTCCTTCCCAAATCTGTCTCCCCAGAAGACACAGGAAACAATGTATTGTCTGCCCAGCAATCAAAGGCAATGCTCAAACACCCAAGTGGCCCCCACCCTCAGCCCGCTCCTGCCCGCCCAGCACCCCCAGGCCCTGGGGGACCTGGGGTTCTCAGACTGCCAAAGAAGCCTTGCCATCTGGCGCTCCCATGGCTCTTGCAACATCTCCCCTTCGTTTTTGAGGGGGTCATGCCGGGGGAGCCACCAGCCCCTCACTGGGTTCGGAGGAGAGTCAGGAAGGGCCACGACAAAGCAGAAACATCGGATTTGGGGAACGCGTGTCAATCCCTTGTGCCGCAGGGCTGGGCGGGAGAGACTGTTCTGTTCCTTGTGTAACTGTGTTGCTGAAAGACTACCTCGTTCTTGTCTTGATGTGTCACCGGGGCAACTGCCTGGGGGCGGGGATGGGGGCAGGGTGGAAGCGGCTCCCCATTTTATACCAAAGGTGCTACATCTATGTGATGGGTGGGGTGGGGAGGGAATCACTGGTGCTATAGAAATTGAGATGCCCCCCCAGGCCAGCAAATGTTCCTTTTTGTTCAAAGTCTATTTTTATTCCTTGATATTTTTCTTTTTTTTTTTTTTTTTTTGTGGATGGGGACTTGTGAATTTTTCTAAAGGTGCTATTTAACATGGGAGGAGAGCGTGTGCGGCTCCAGCCCAGCCCGCTGCTCACTTTCCACCCTCTCTCCACCTGCCTCTGGCTTCTCAGGCCTCTGCTCTCCGACCTCTCTCCTCTGAAACCCTCCTCCACAGCTGCAGCCCATCCTCCCGGCTCCCTCCTAGTCTGTCCTGCGTCCTCTGTCCCCGGGTTTCAGAGACAACTTCCCAAAGCACAAAGCAGTTTTTCCCCCTAGGGGTGGGAGGAAGCAAAAGACTCTGTACCTATTTTGTATGTGTATAATAATTTGAGATGTTTTTAATTATTTTGATTGCTGGAATAAAGCATGTGGAAATGACCCAAA |
| --- |

Sequence marked in bright: The specific sequences for fluorescent probe targeting.

**Table S4 Sequences employed in targeted *COL1A1* mRNA and other mRNA**

| Number | Name | Sequence 5’-3’ |
| --- | --- | --- |
| 1 | T1 | AGG UAC CAU GAC CGA GAC GUG UGG A |
|  | P1-1 | U CCA CAC GUCUACGUACGAAGGAGAGGAGA |
|  | P2-1 | GGUAGAGGAGAGUACGCAUCAUGGUACCU |
| 2 | T2 | U CCC UGG CGA GCG UGG UGU GCA AG |
|  | P1-2 | CUUG CAC ACC AA CGUACGAAGGAGAGGAGA |
|  | P2-2 | GGUAGAGGAGAGUACG CA UC GCC AGG GA |
| 3 | T3 | CAAGGACAAGAGGCAUGUCUGGUUCG |
|  | P1-3 | CAAGGACAAGAAACGUACGAAGGAGAGGAGA |
|  | P2-3 | GGUAGAGGAGAGUACG AC UGUCUGGUUC |
|  | *ACTA2* | TGTCTCTCTATGCCTCTGGACGCAC |
|  | *COL3A1* | TTGGTCAGTCCTATGCGGATAGAGA |
|  | *FN1* | TTGGTGGCAACTTGCCTCCCGGTGC |
|  | *COL1A2* | GCGCCCGCCAGGTGATACCTCCGCC |

**Table S5 Sequences for colocalization experiment**

| Group | Name | Sequence 5’-3’ |
| --- | --- | --- |
| Target | T4 | UGGGGAACGCGUGUCAAUCCCUUGU |
| Fixed cells | Fish | Cy3-ACA AGG GAT TGA CAC GCG TTC CCC A |
| Living cells | ipepper | ACAAGGGAUGGCACUGGCGCCGAGGGCUUCCUCCAAUCGUGGCGUGUCGGCCACACGCGUUCCCCA |

**Table S6 Name of natural compounds in high throughput screening experiment**

| **No.** | **Name** | **No.** | **Name** | **No.** | **Name** |
| --- | --- | --- | --- | --- | --- |
| 1 | Astragaloside | 15 | Notoginseng Total Saponins | 29 | Gallic Acid |
| 2 | Ginkgolide B | 16 | Tanshinone Acid B | 30 | Ginsenoside PF |
| 3 | Berberine | 17 | Dipsacoside IV | 31 | Isochlorogenic Acid A |
| 4 | Alpha-Hedera Saponin | 18 | Tanshinone Acid A | 32 | Dipsacoside B |
| 5 | Isochlorogenic Acid C | 19 | Dihydrotanshinone I | 33 | Salicylic Acid |
| 6 | Epimedin B | 20 | Ginsenoside Rb1 | 34 | Ginsenoside Rg3 |
| 7 | Puerarin | 21 | Icariin | 35 | Ginkgolide C |
| 8 | Tanshinone IIA | 22 | Ginsenoside Rg5 | 36 | Kaempferol |
| 9 | Saikosaponin A | 23 | Curcumin | 37 | Ginsenoside Rk3 |
| 10 | Quercetin | 24 | Genistein | 38 | Chlorogenic Acid |
| 11 | Lycopene | 25 | Glycyrrhizic Acid | 39 | Genistin |
| 12 | Oleanolic Acid | 26 | Catecholaldehyde | 40 | Epimedin B |
| 13 | Rhodiola Saccharide | 27 | Ginsenoside Rg1 | 41 | Dahurian Cimicifuga Rhizome |
| 14 | Saikosaponin Glucoside | 28 | 3-Hydroxy Puerarin | 42 | Cryptotanshinone |

**Table S7 Primers for** **RT-qPCR**

| **Gene** | **Name** | **Sequence 5’-3’** |
| --- | --- | --- |
| Human *FN* | Forward | AGCCGAGGTTTTAACTGCGA |
|  | Reverse | CCCACTCGGTAAGTGTTCCC |
| Human *ACTA2* | Forward | CTATGAGGGCTATGCCTTGCC |
|  | Reverse | GCTCAGCAGTAGTAACGAAGGA |
| Human *COL1A1* | Forward | TGCTCGTGGAAATGATGGTG |
|  | Reverse | GGAGCACCATTGGCACCTTT |
| Mice *Col1a1* | Forward | TGCTAACGTGGTTCGTGACCGT |
|  | Reverse | ACATCTTGAGGTCGCGGCATGT |
| Mice *Acta2* | Forward | GGCACCACTGAACCCTAAGG |
|  | Reverse | ACAATACCAGTTGTACGTCCAGA |
| Mice *Fn* | Forward | CCCTATCTCTGATACCGTTGTCC |
|  | Reverse | TGCCGCAACTACTGTGATTCGG |
| β-actin | Forward | AGGATTCCTATGTGGGCGAC |
|  | Reverse | ATAGCACAGCCTGGATAGCAA |
| Probe 1-1 | Forward | CCACACGTCTACGTACGAAGGAG |
|  | Reverse | AGTGCAGGGTCCGAGGTATT |

**Table S8 Antibodies for immunoblotting**

| Antibody | Company | Catalog # |
| --- | --- | --- |
| COL1A1 | Cell Signaling Technology | 91144S |
| α-SMA | Cell Signaling Technology | 19245S |
| FN | Abcam | Ab2413 |
| GAPDH | Proteintech | 60004-1-Ig |

1. **Figures**


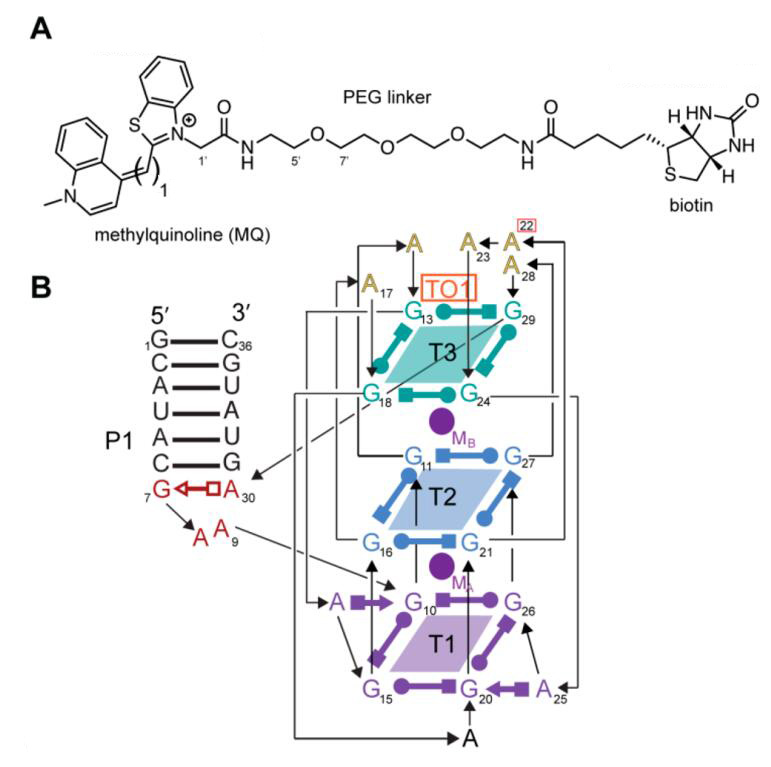


**Figure S1** The structure of TO1-Biotin and RNA Mango Ⅱ.^[2]^


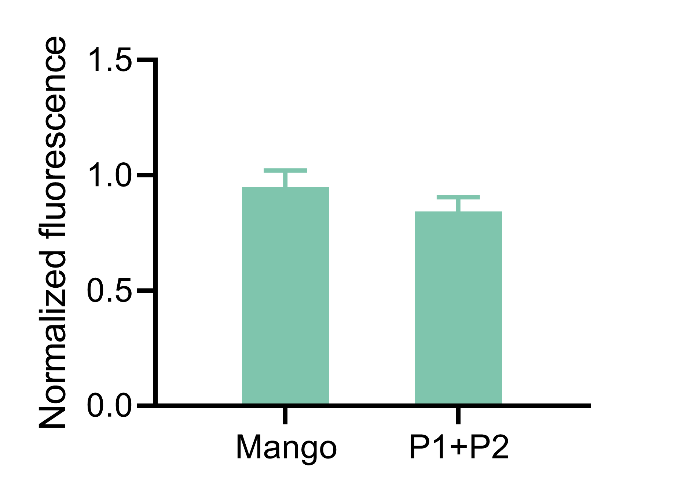


**Figure S2** Fluorescence intensity of RNA Mango Ⅱ, P1and P2 after T recognition in 40 mM HEPES, 5 mM MgCl_2_, 100 mM KCl, 10 μM TO1-Biotin.


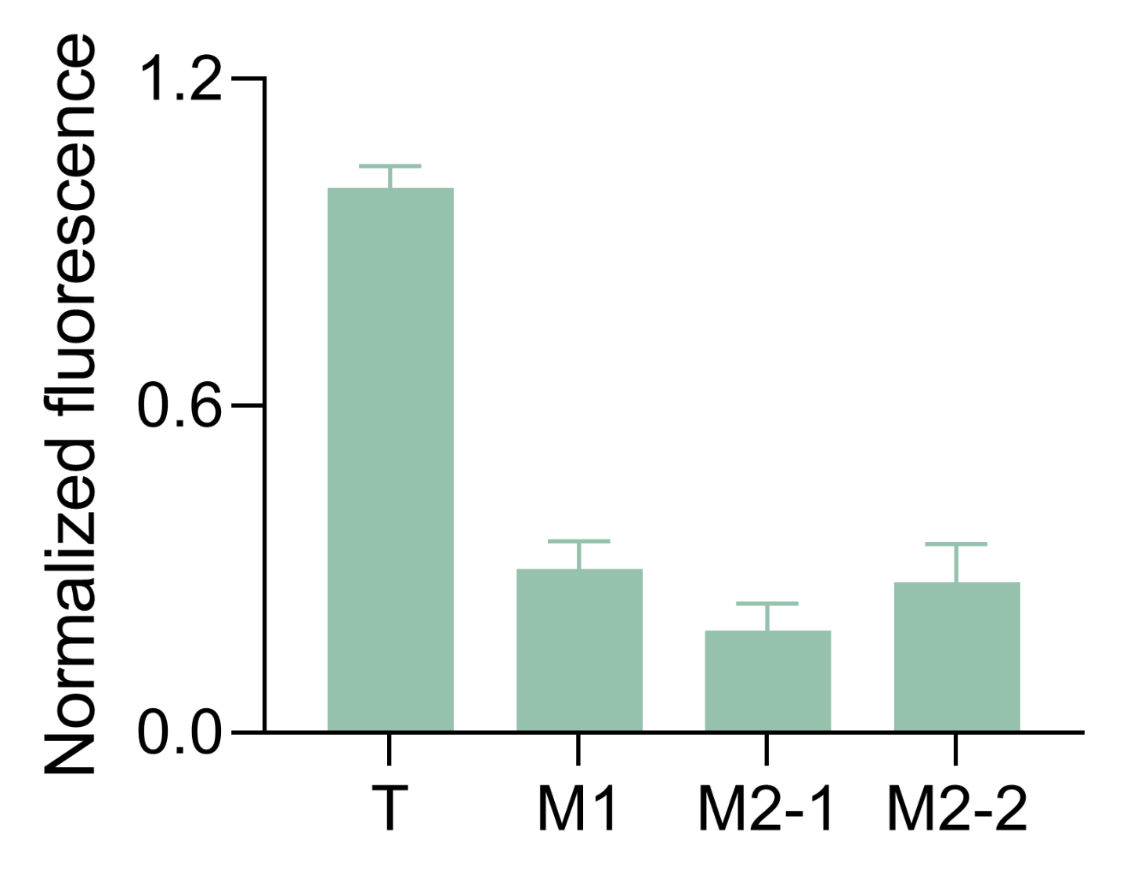


**Figure S3** Fluorescence corresponding to T incubated with P1 and P2 in HEPES buffer (40 mM, pH 7.4) containing 10 µM TO1-Biotin, 100 mM KCl, and 5 mM MgCl₂.1 μM T (perfect match); 1 μM one base mismatch(M1); 1 μM two bases mismatch at site 1 (M2-1); 1 μM two bases mismatch at site 2 (M2-2).


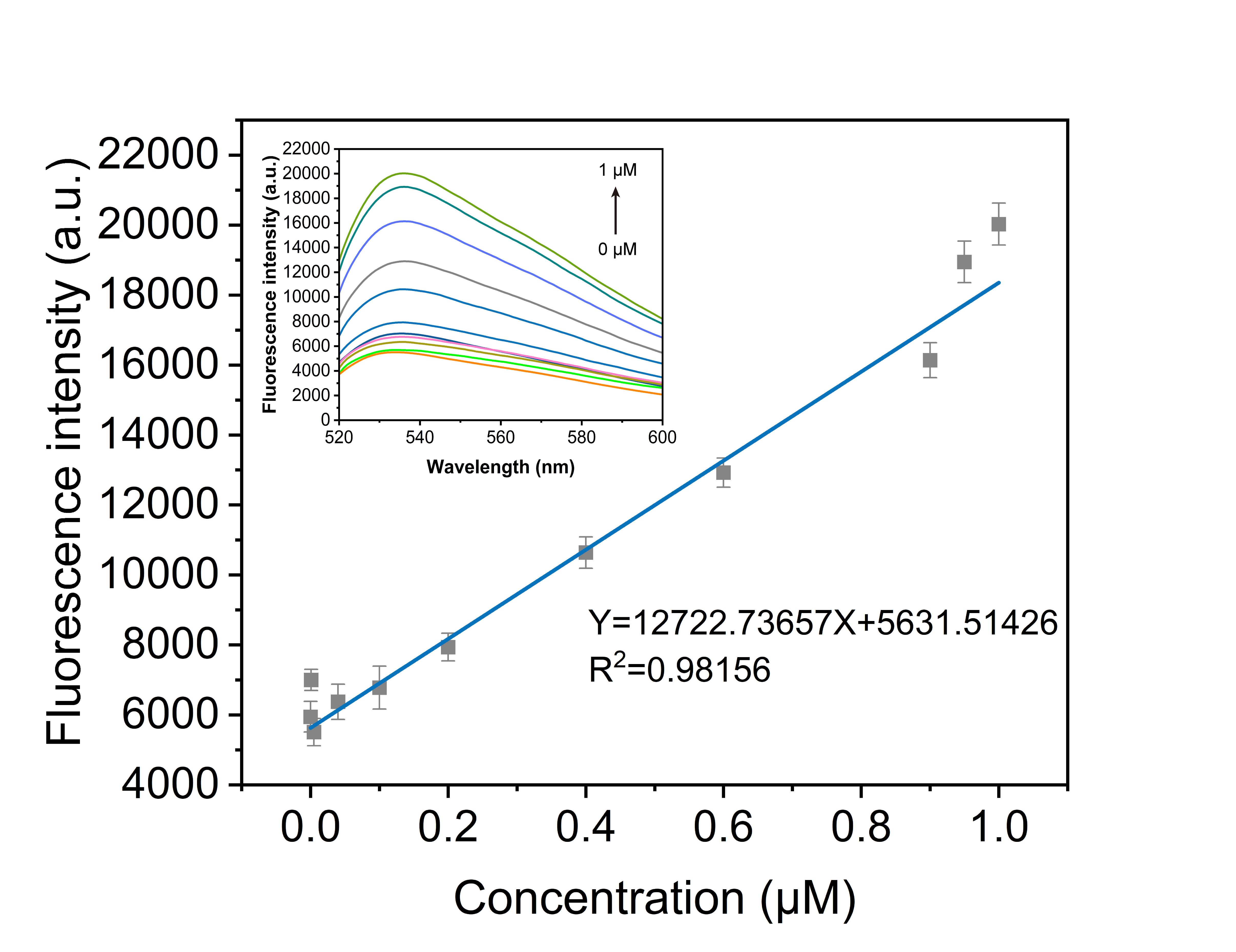


**Figure S4** Fluorescence corresponding to different concentrations (0.001, 0.005, 0.04, 0.1, 0.2, 0.4, 0.6, 0.9, 0.95, 1.0 µM) target incubated with 1 μM probe 1 and probe 2 in HEPES buffer (40 mM, pH 7.4) containing 10 µM TO1-Biotin, 5 mM MgCl_2_, 100 mM KCl. Inset: Calibration curve of fluorescence intensity vs. T concentration.





**Figure S5** Circular dichroism spectra of RNA Mango II and target incubated with 1 μM probe 1 and probe 2 in HEPES buffer (40 mM, pH 7.4) containing 10 µM TO1-Biotin, 5 mM MgCl_2_, 100 mM KCl.

**Figure S6** The *COL1A1* mRNA expression levels of LX-2 cells treated with 10 ng mL^-1^ TGF-β for 0 h, 6 h, 12 h, 18 h or 24 h (n = 6). ^**^ *p*<0.01, ^***^ *p*<0.001, ^****^ *p*<0.0001. NS indicates no statistical significance. Bars represent mean ± SEM values. Statistical differences were determined by one-way ANOVA.


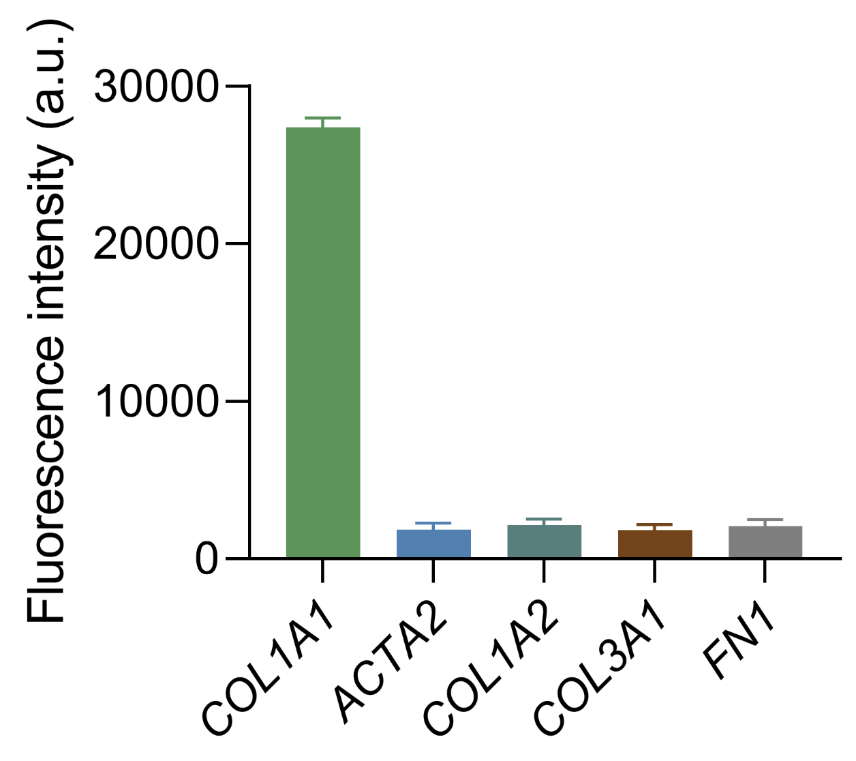


**Figure S7** Fluorescence corresponding to incubated different mRNA specific sequences (concentration of *COL1A1*: 1µM, others’ concentration:5 µM, n = 3) with our probes in HEPES buffer (40 mM, pH 7.4) containing 10 µM TO1-Biotin, 100 mM KCl, and 5 mM MgCl₂.


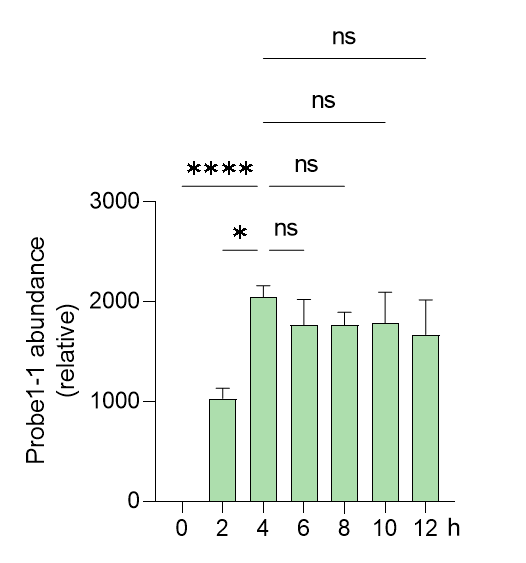


**Figure S8** The abundance of probe 1-1 in LX-2 cells after transfecting for 0 h, 2 h, 4 h, 6 h, 8 h, 10 h or 12 h by RT-qPCR analysis (n = 4). ^*^ *p*<0.05, ^****^ *p*<0.0001. NS indicates no statistical significance. Bars represent mean ± SEM values. Statistical differences were determined by one-way ANOVA.

The results (Figure S8) indicated that after transfection, the probe experienced certain degree of degradation due to the cellular environment; however, it generally maintained a stable level within the 12-h period. Given that the amount of probe used was significantly in excess relative to the mRNA content, the effects of the cellular environment and RNA degradation are acceptable under the requirements of our experiment.


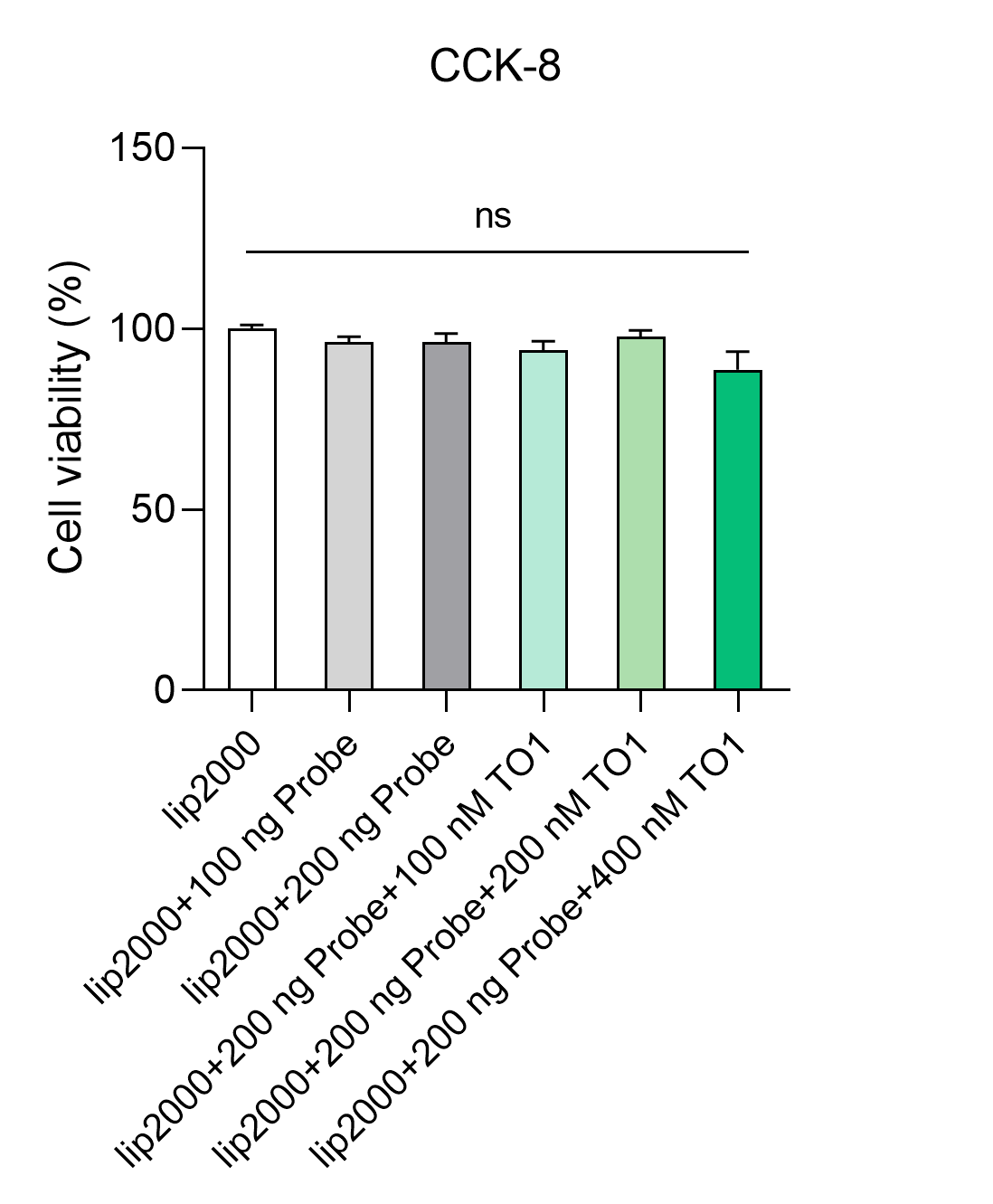


**Figure S9** The probes and TO1-Biotin had no effect on LX-2 cells viability. LX-2 cells were activated by TGF-β for 24 h after seeding in the plate. The cells were transfected with different concentrations (100 ng, 200 ng) of probes and incubated with different concentrations (100 nM, 200 nM, 400 nM) of TO1-Biotin. NS indicates no statistical significance. Bars represent mean ± SEM values. Statistical differences were determined by one-way ANOVA.


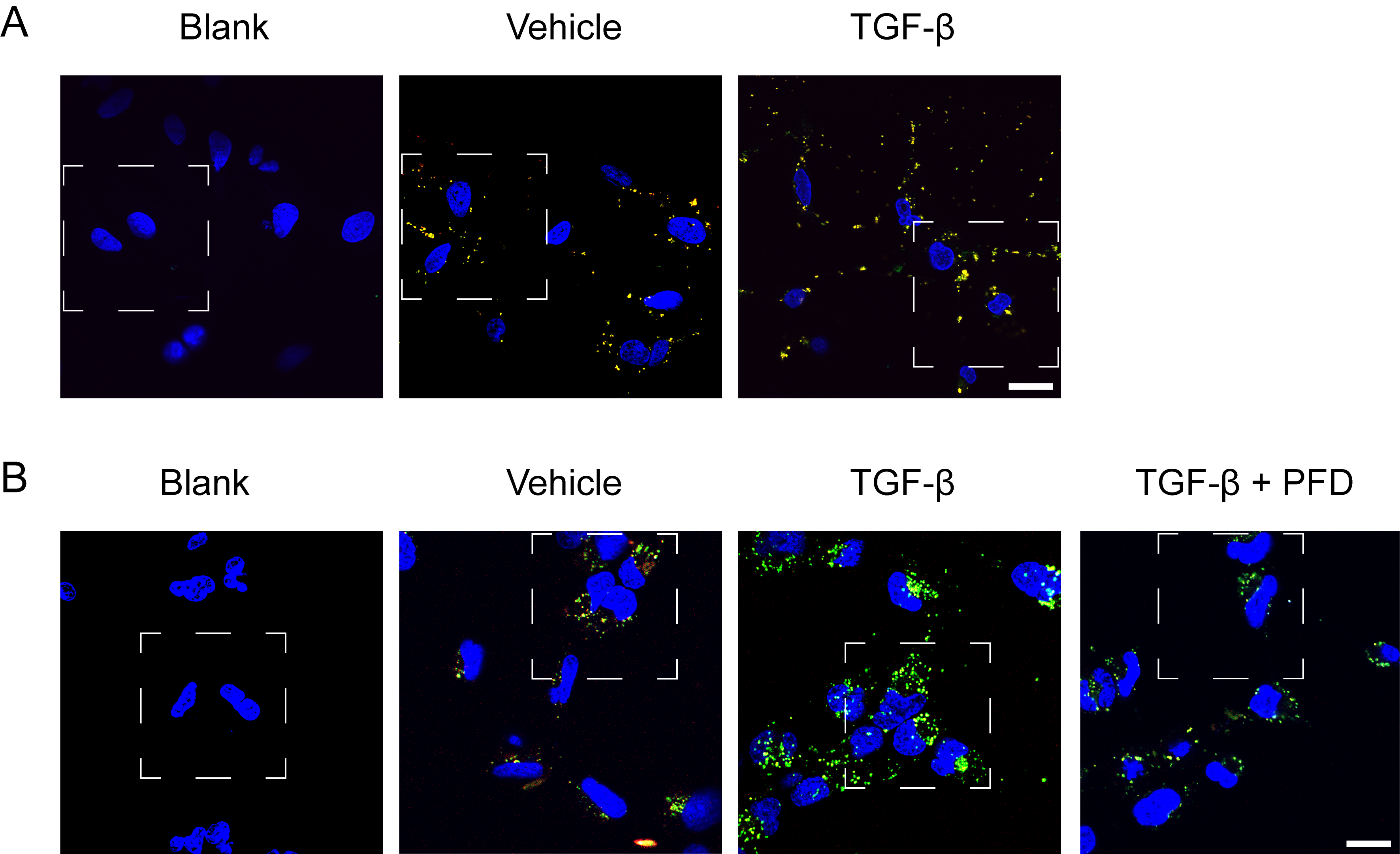


**Figure S10** (A) Fixed-cell co-localization confocal images of transcribed probes with TO1-Biotin (green) and Cy3-labeled FISH (red) targeted *COL1A1* mRNA in LX-2 cells. Scale bar = 10 µm. (B) Live cells co-localization confocal images of transcribed probes with TO1-Biotin (green) and ipepper-HBC620 (red) targeted *COL1A1* mRNA in LX-2 cells. Scale bar = 10 µm. Due to the large size of the figure, we have only included a portion of it in the main text to ensure clarity.


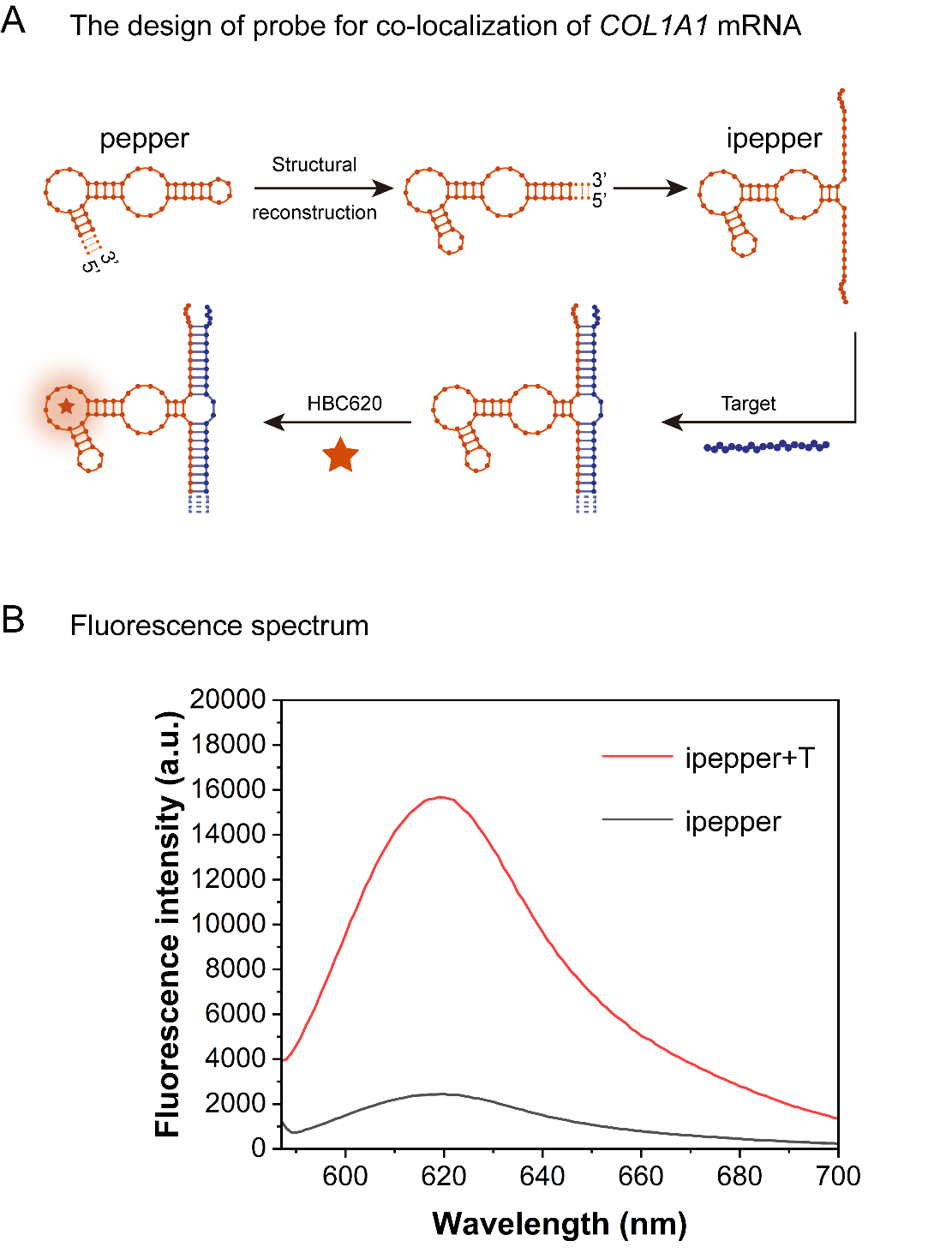


**Figure S11** (A) The design process of pepper-based probes (ipepper) for the colocalization of *COL1A1* mRNA in live LX-2 cells (B) Fluorescence emission spectrum of probes excited at 562 nm after incubating with or without T4 in HEPES buffer (40 mM, pH 7.4) containing 10 μM TO1-Biotin, 100 mM KCl, and 10 mM MgCl_2_.

Based on previous reports,^[3]^ loop 1 (L1) and loop 2 (L2) of the Pepper aptamer are critical for HBC dye binding and fluorescence activation (Figure 1). To improve the conformational stability of L1, we introduced an auxiliary loop (L3) adjacent to stem 1 (S1), which significantly enhances local folding. Optimization of stem 2 (S2) length revealed that stems shorter than 4 base pairs failed to form stable structures, and constructs with only 3 bp showed no significant fluorescence upon HBC620 binding. This indicates that a minimum of 3 bp is essential to maintain the functional structure of L2. Accordingly, the length of S2 was fixed at 3 bp, and a T4-specific recognition sequence was incorporated to construct a T4-triggered aptamer reassembly probe (iPepper). Fluorescence spectroscopy validated the design: in the absence of T4, the iPepper–HBC620 complex emitted only weak background signals, whereas T4 recognition induced a pronounced fluorescence enhancement at 620 nm. These results demonstrate the probe's high specificity for T4 and its suitability for fluorescence colocalization imaging of endogenous *COL1A1* mRNA.


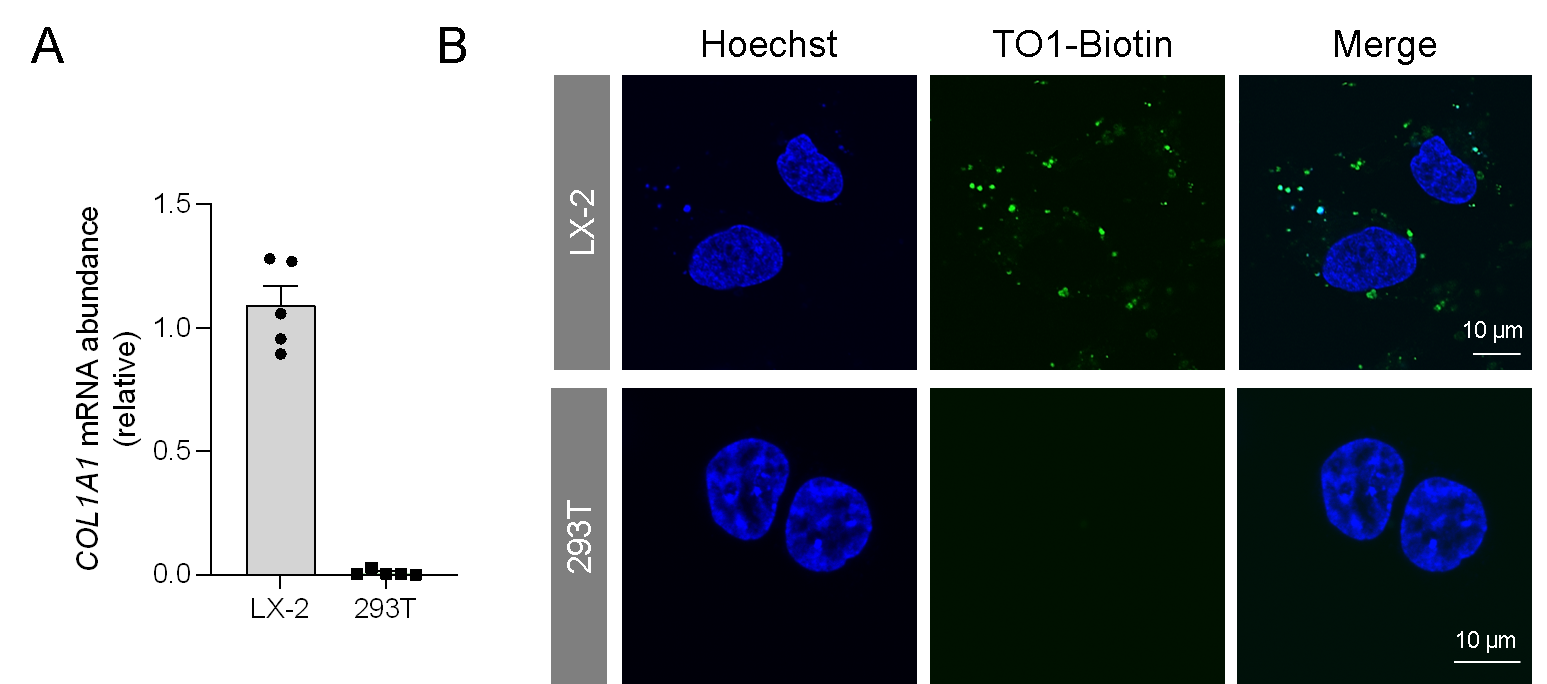


**Figure S12** (A) The *COL1A1* mRNA expression levels of LX-2 and HEK-293T cells by RT-qPCR analysis. Bars represent mean ± SEM values. Statistical differences were determined by *t* test. (B) Confocal images of LX-2 and HEK-293T cells transfected with probes targeting endogenous *COL1A1* mRNA. Scale bar = 10 µm.


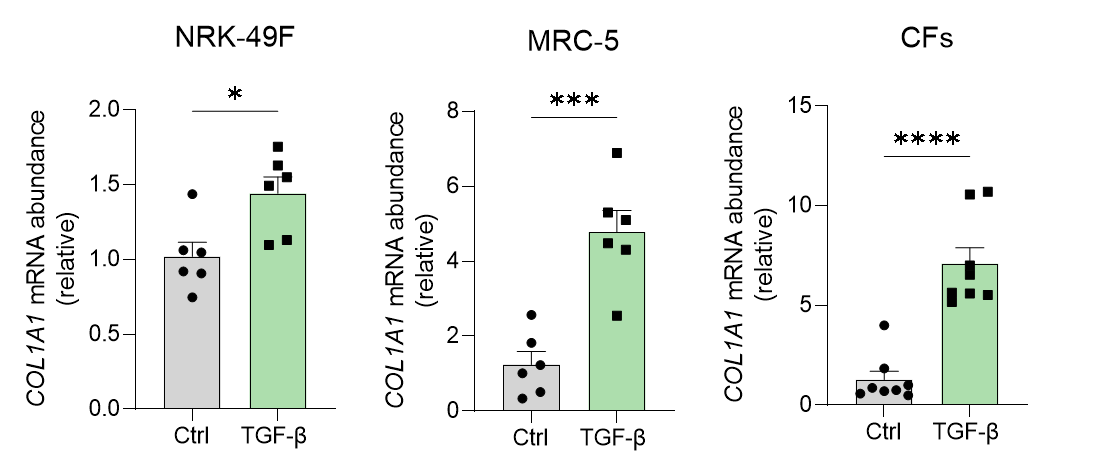


**Figure S13** The *COL1A1* mRNA expression levels of kidney fibroblasts (NRK-49F), lung fibroblasts (MRC-5), lung fibroblasts (MRC-5) treated with 10 ng mL^-1^ TGF-β for 24 h by RT-qPCR analysis. ^*^ *p*<0.05, ^***^ *p*<0.001, ^****^ *p*<0.0001. Bars represent mean ± SEM values. Statistical differences were determined by *t* test.


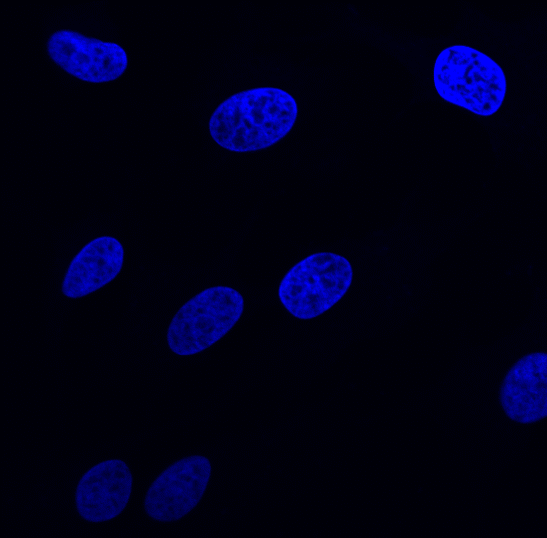

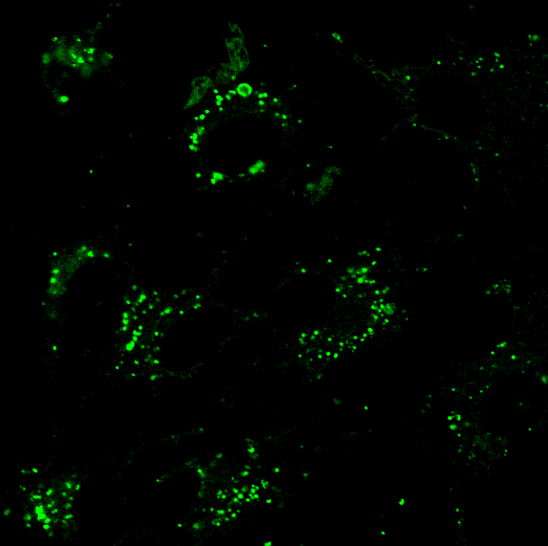

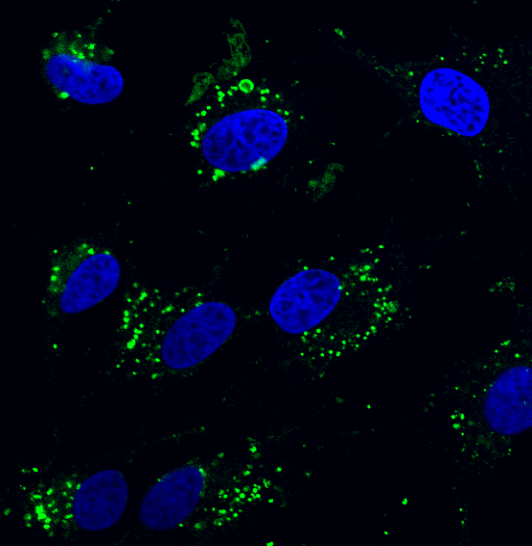

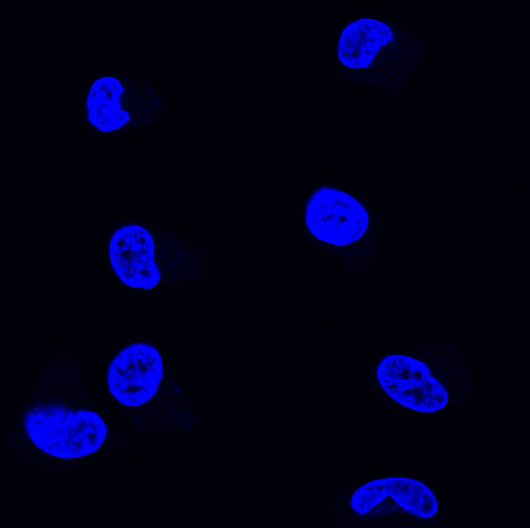

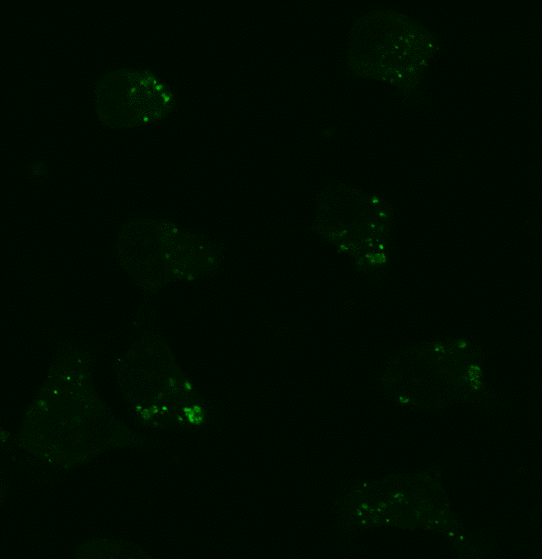

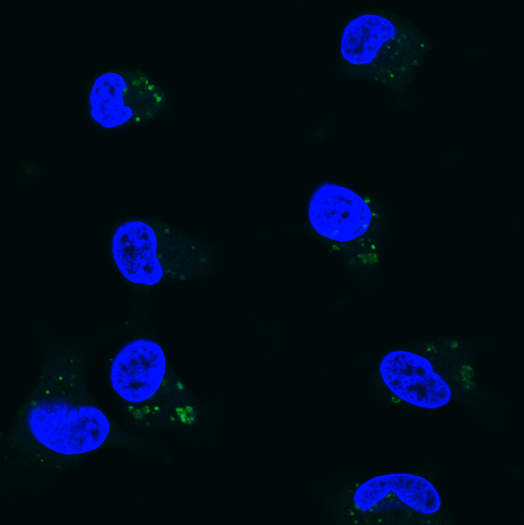


Vehicle

TGF-β


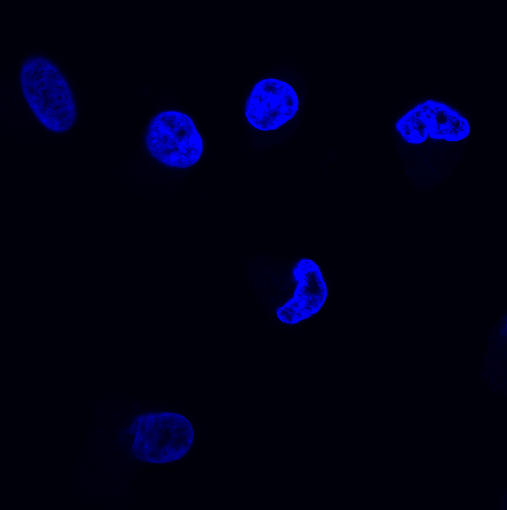

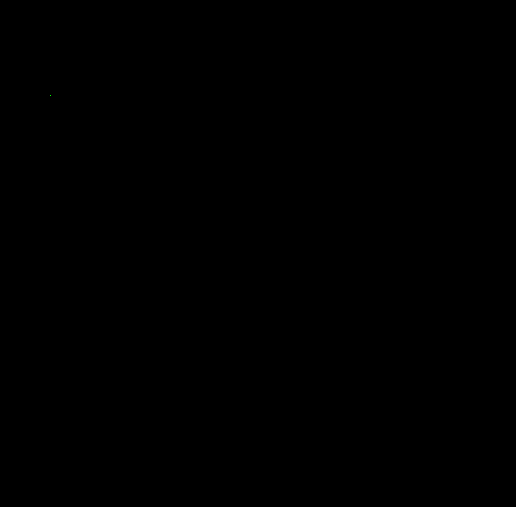

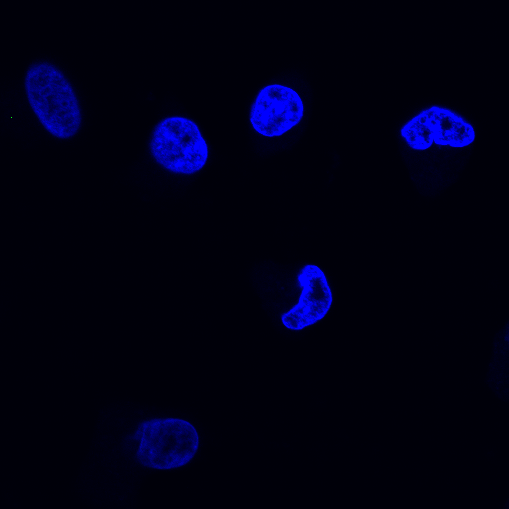


Blank

**Figure S14** Confocal images of kidney fibroblasts (NRK-49F) transfected with probes targeting endogenous *COL1A1* mRNA. Blank means not transfected with probes. Scale bar = 10 µm.


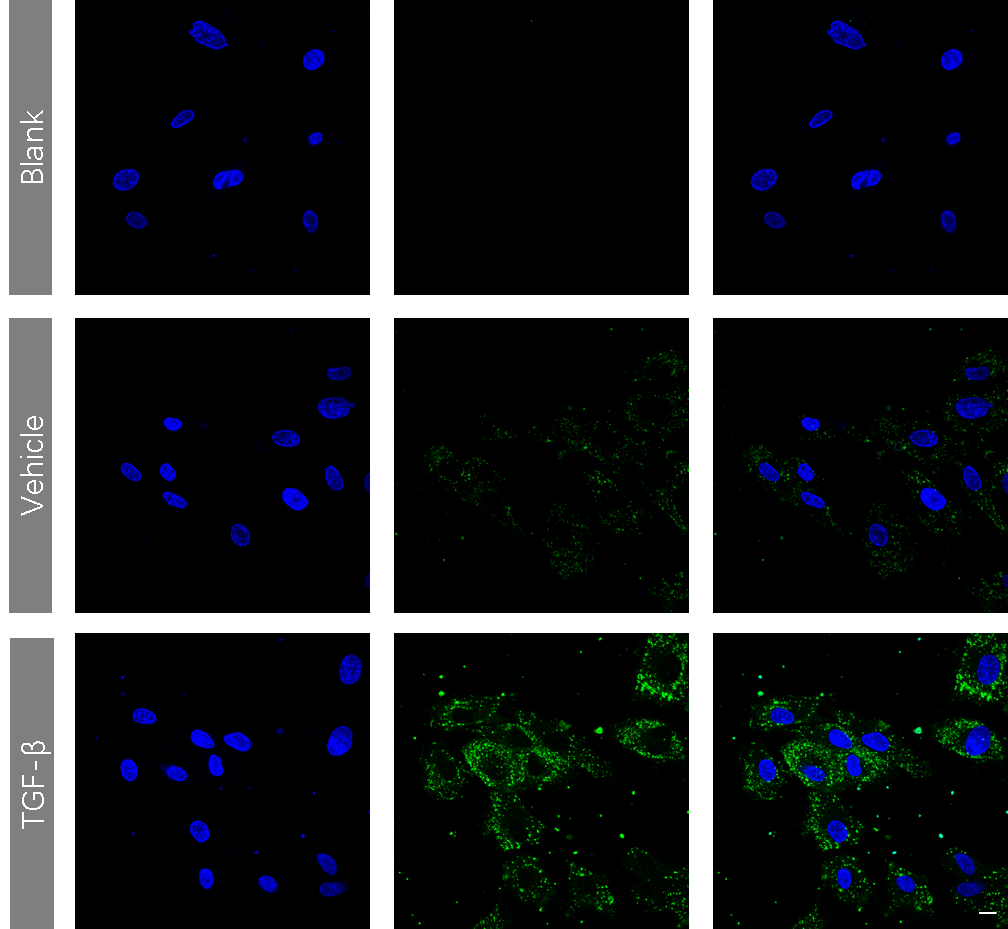


**Figure S15** Confocal images of lung fibroblasts (MRC-5) transfected with probes targeting endogenous *COL1A1* mRNA. Blank means not transfected with probes. Scale bar = 10 µm.


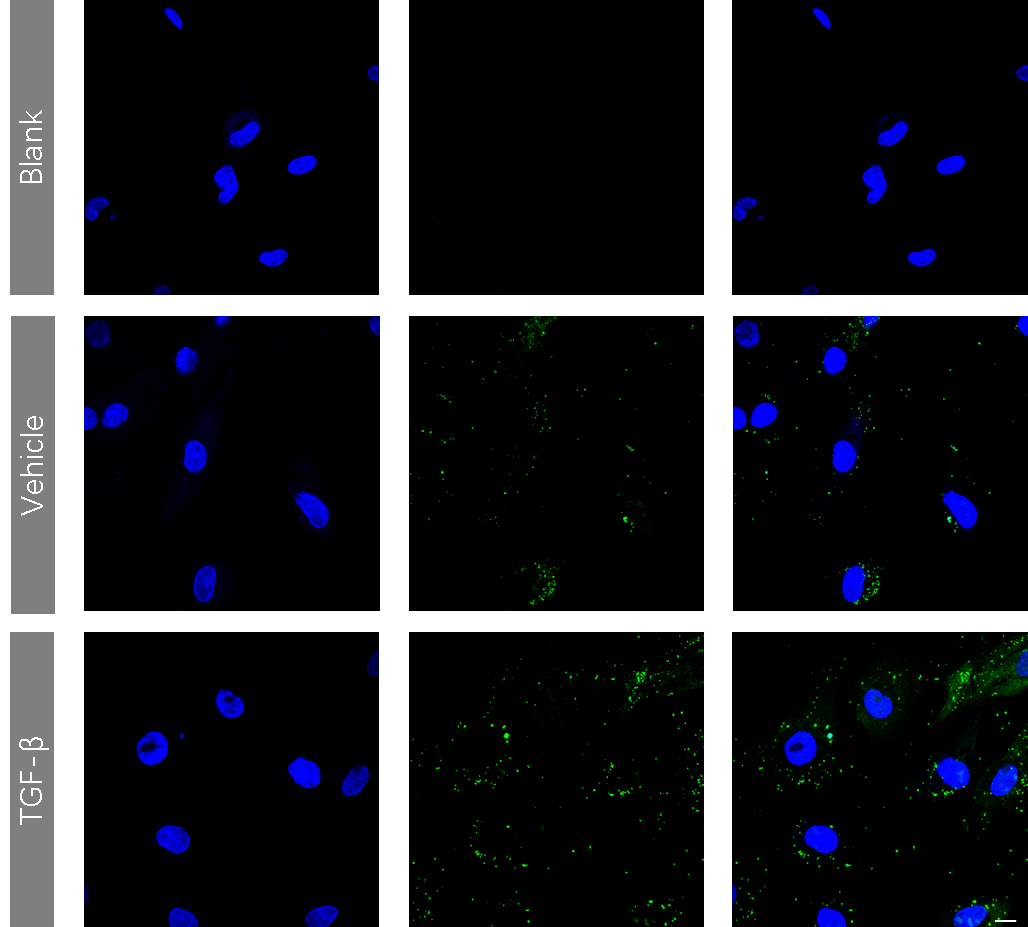


**Figure S16** Confocal images of cardiac fibroblasts (CFs) transfected with probes targeting endogenous *COL1A1* mRNA. Blank means not transfected with probes. Scale bar = 10 µm.


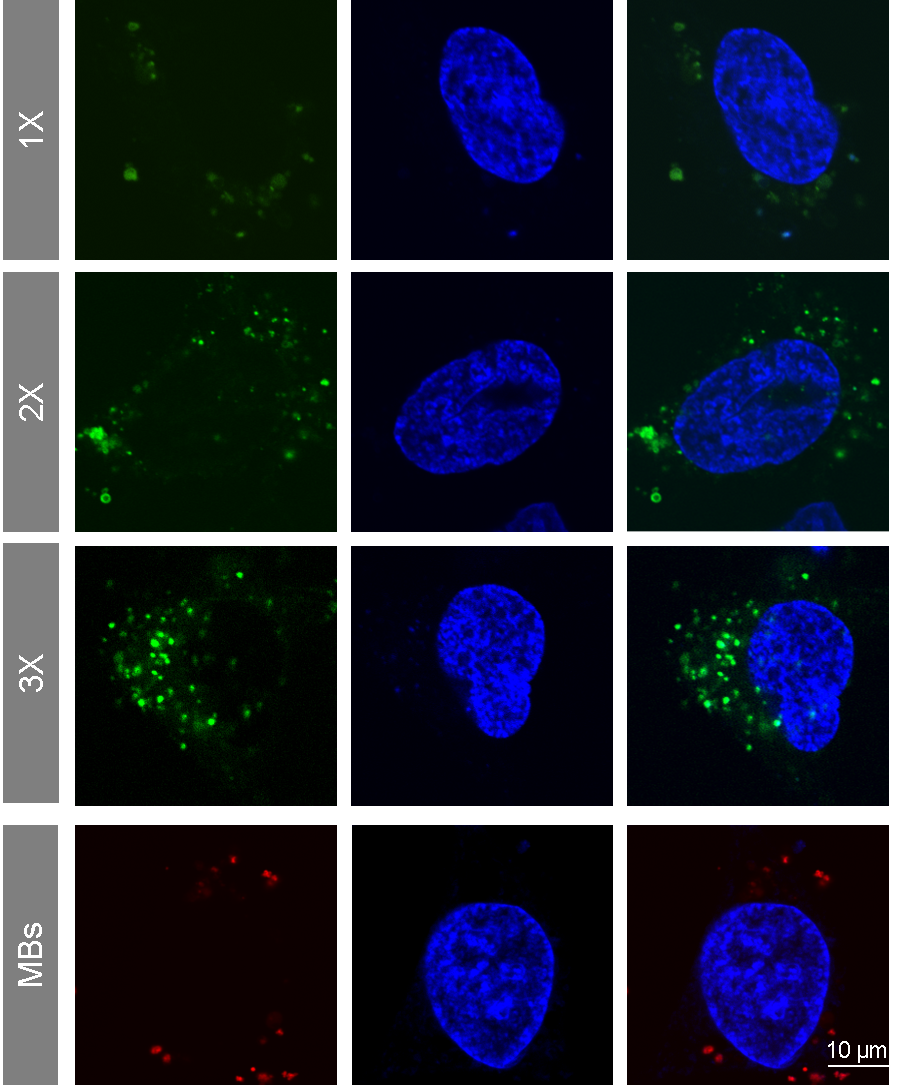


**Figure S17** Fluorescence images of the number of probes groups (1×, 2× and 3×) and MBs for *COL1A1* mRNA labeling with TO1-Biotin.


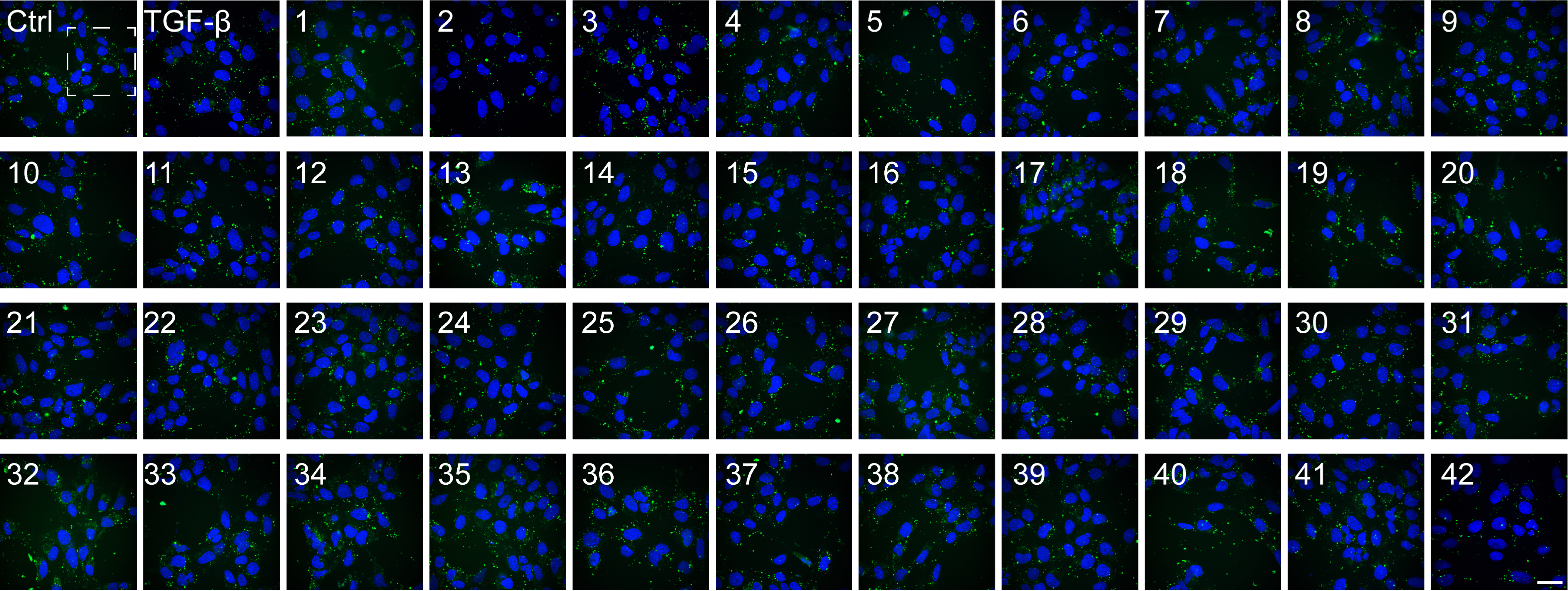


**Figure S18** LX-2 cells were stimulated with 10 ng mL^-1^ TGF-β and treated with 10 μM of various natural compounds (n = 42) for 24 h, respectively. High throughput live-cell imaging was performed, with green fluorescence indicating *COL1A1* mRNA expression levels. Scale bar = 50 µm. Due to the large size of the figure, we have only included a portion of it in the main text to ensure clarity.

**
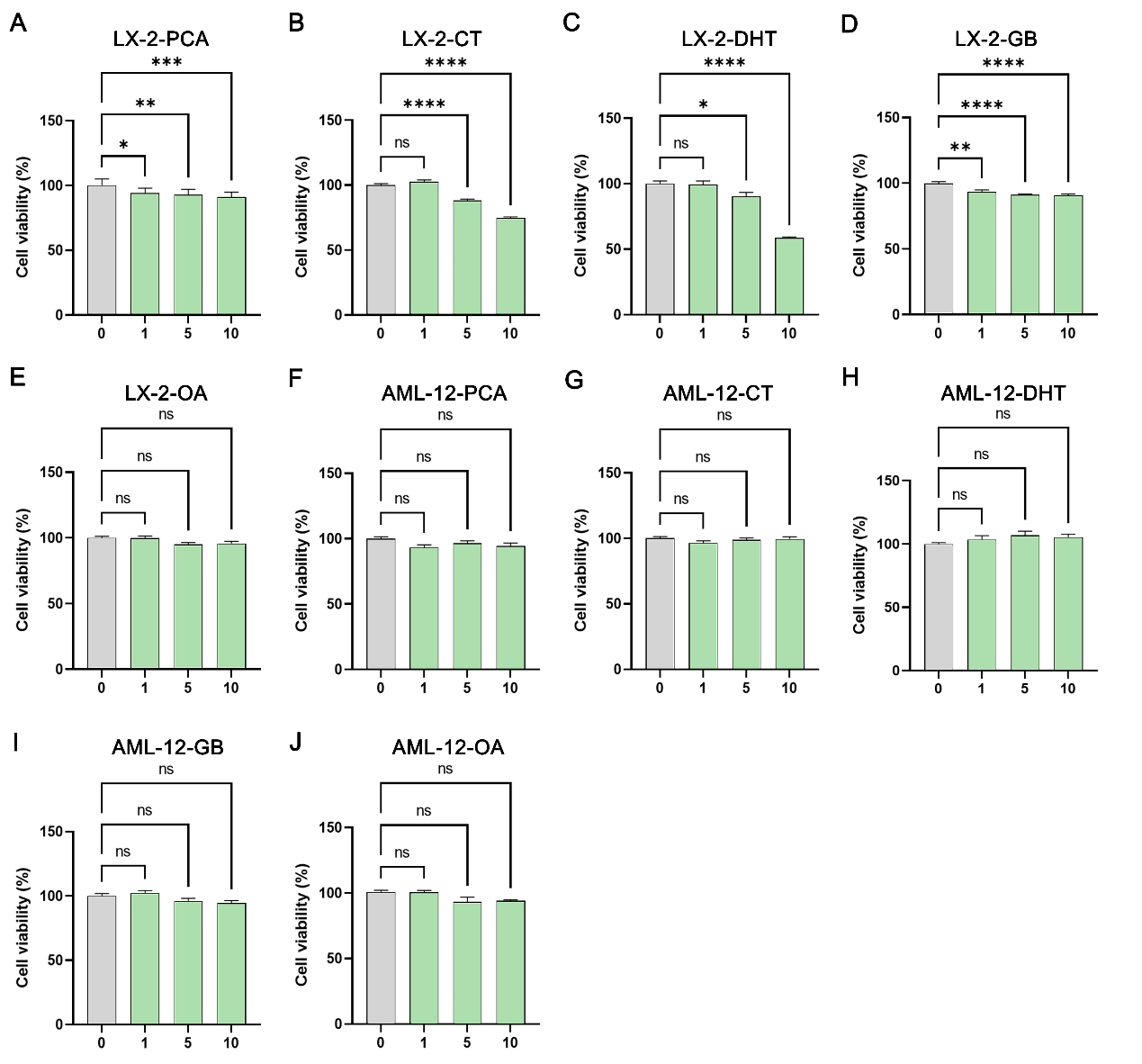
**

**Figure S19** Effects of different concentrations of compounds on hepatic stellate cells (LX-2) and normal hepatocytes (AML-12). (A-E) LX-2 cells were treated with different concentrations (0 μM, 1 μM, 5 μM, 10 μM) of PCA (A), CT (B), DHT (C), GB (D), or OA (E) for 24 h. (F-J) AML12 cells were treated with different concentrations (0 μM, 1 μM, 5 μM, 10 μM) of PCA (F) or CT (G) or DHT (H) or GB (I) or OA (J) for 24 h. ^*^ *p*<0.05, ^**^ *p*<0.01, ^***^ *p*<0.001, ^****^ *p*<0.0001. NS indicates no statistical significance. Bars represent mean ± SEM values. Statistical differences were determined by one-way ANOVA.


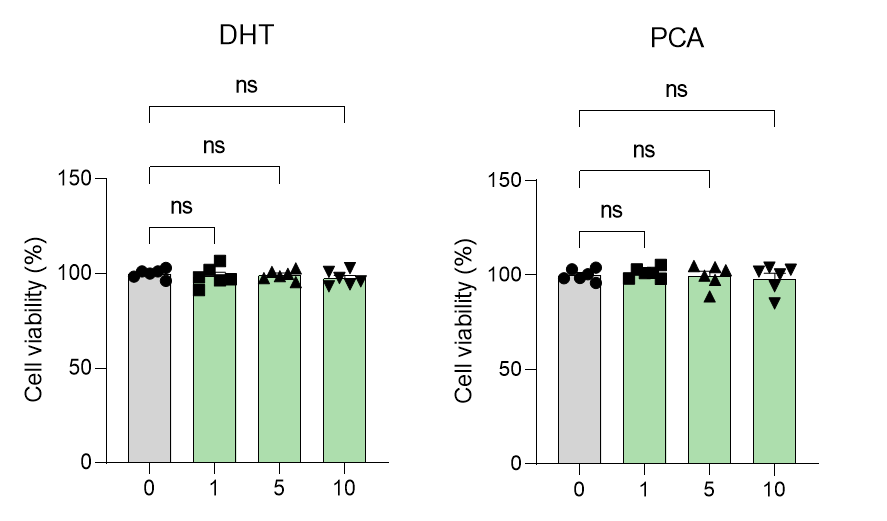


**Figure S20** The cell viability assay demonstrated that DHT and PCA had no significant impact on the viability of LX-2 cells within 2 h. LX-2 cells were treated with different concentrations (0 μM, 1 μM, 5 μM, 10 μM) of DHT (A) or PCA (B) for 2 h. NS indicates no statistical significance. Error bars represent mean ± SEM values. Statistical differences were determined by one-way ANOVA. Source data are provided as a Source file.


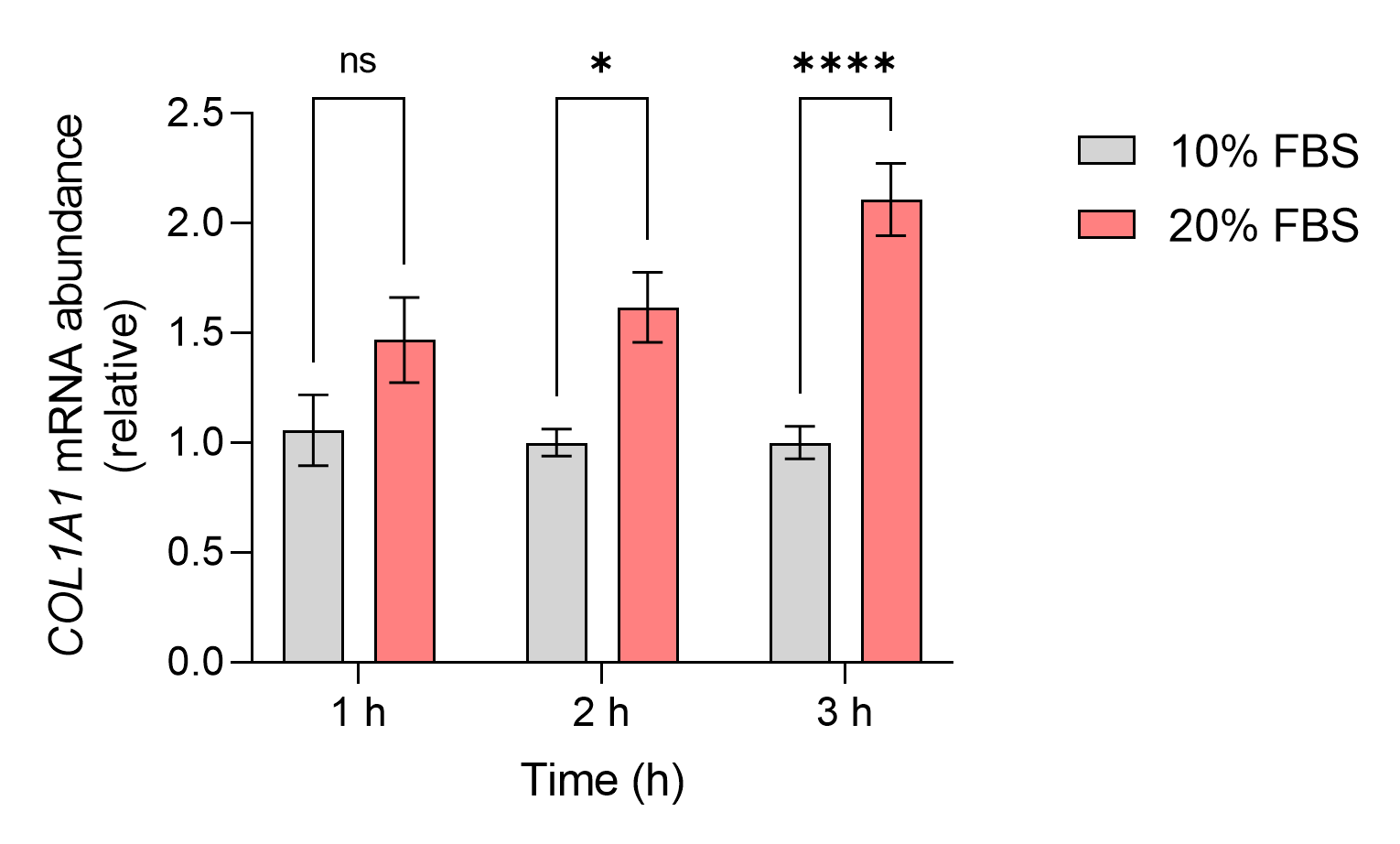


**Figure S21** LX-2 cells were significantly activated by using 20% FBS within 3 h. LX-2 cells were transfected with the probe for 4 h, and the cells were then treated with 10% FBS or 20% FBS. RT-qPCR analyses of *COL1A1* mRNA levels in LX-2 cells after treatment with FBS. NS indicates no statistical significance. ^*^ *p*<0.05, ^****^ *p*<0.001. Bars represent mean ± SEM values; statistical differences were determined by two-way ANOVA.

**References**

[1] E. V. Dolgosheina, S. C. Jeng, S. S. Panchapakesan, R. Cojocaru, P. S. Chen, P. D. Wilson, N. Hawkins, P. A. Wiggins, P. J. Unrau, RNA Mango Aptamer-Fluorophore: A Bright, High-Affinity Complex for RNA Labeling and Tracking, ACS Chem. Biol. 2014, 9, 2412.

[2] R. J. Trachman, A. Abdolahzadeh, A. Andreoni, R. Cojocaru, J. R. Knutson, M. Ryckelynck, P. J. Unrau, A. R. Ferre-D'Amare, Crystal Structures of the Mango-II RNA Aptamer Reveal Heterogeneous Fluorophore Binding and Guide Engineering of Variants with Improved Selectivity and Brightness, Biochemistry 2018, 57, 3544.

[3] Q. Wang, F. Xiao, H. Su, H. Liu, J. Xu, H. Tang, S. Qin, Z. Fang, Z. Lu, J. Wu, X. Weng, X. Zhou, Inert Pepper Aptamer-Mediated Endogenous mRNA Recognition and Imaging in Living Cells, Nucleic Acids Res. 2022, 50, e84.
